# Missing Data Gap Imputation Methods in Electroencephalogram (EEG) Signals: A Systematic Scoping Review

**DOI:** 10.3390/s26082431

**Published:** 2026-04-15

**Authors:** Tobias Bergmann, Michael Movshovich, Yushu Shao, Julia Ryznar, Xue Nemoga-Stout, Izabella Marquez, Isuru Herath, Amanjyot Singh Sainbhi, Nuray Vakitbilir, Noah Silvaggio, Rakibul Hasan, Kevin Y. Stein, Hina Shaheen, Jaewoong Moon, Frederick A. Zeiler

**Affiliations:** 1 Graduate Program in Biomedical Engineering, Price Faculty of Engineering, University of Manitoba, Winnipeg, MB R3T 5V6, Canada; herathi1@myumanitoba.ca (I.H.); amanjyot.s.sainbhi@gmail.com (A.S.S.); vakitbir@myumanitoba.ca (N.V.); hasanr2@myumanitoba.ca (R.H.); steink34@myumanitoba.ca (K.Y.S.);; 2 Undergraduate Medicine, Rady Faculty of Health Sciences, University of Manitoba, Winnipeg, MB R3E 3P5, Canada; movshovm@myumanitoba.ca; 3 Department of Statistics, Faculty of Science, University of Manitoba, Winnipeg, MB R3T 2M8, Canada; shaoy4@myumanitoba.ca (Y.S.); hina.shaheen@umanitoba.ca (H.S.); 4 Undergraduate Engineering, Price Faculty of Engineering, University of Manitoba, Winnipeg, MB R3T 5V6, Canada; ryznarj@myumanitoba.ca (J.R.); nemogasx@myumanitoba.ca (X.N.-S.); marquezi@myumanitoba.ca (I.M.); 5 Department of Human Anatomy and Cell Science, Rady Faculty of Health Sciences, University of Manitoba, Winnipeg, MB R3E 0J9, Canada; 6 Section of Neurosurgery, Department of Surgery, Rady Faculty of Health Sciences, University of Manitoba, Winnipeg, MB R3A 1R9, Canada; 7 Department of Clinical Neuroscience, Karolinska Institutet, 171 77 Stockholm, Sweden; 8 Centre on Aging, University of Manitoba, Winnipeg, MB R3T 2N2, Canada; 9 Division of Anaesthesia, Department of Medicine, Addenbrooke’s Hospital, University of Cambridge, Cambridge CB2 0QQ, UK; 10 Pan Am Clinic Foundation, Winnipeg, MB R3M 3E4, Canada

**Keywords:** electroencephalogram, imputation methodologies, missing segments, gap reconstruction

## Abstract

**Objective:** Electroencephalogram (EEG) measures electrophysiological activity in the cerebral cortex and is broadly used across diagnostic, research, and clinical contexts. Missing data gaps are a pervasive issue in EEG signal recording, resulting from sensor failures and sensor disconnections, amongst other sources. To preserve a continuous signal describing underlying electrophysiological processes, imputation must be used to reconstruct these gaps. The aim of this review is to examine the methods that have been developed for missing data gap imputation in EEG signals. **Methods:** A search of five databases was conducted based on the Preferred Reporting Items for Systematic Reviews and Meta-Analysis guidelines. The search question examined existing algorithms for imputation in EEG signals. **Results:** The initial search yielded 17,490 results (an update included 1913 additional results). This review includes 16 articles presenting EEG gap imputation methods. These imputation methods were characterized as (i) tensor-based, (ii) machine learning and deep learning, and (iii) model-based and classical. **Conclusions:** Several of these methods achieved strong effectiveness for accurately reconstructing gaps in ‘ground truth’ EEG signals; however, the limited generalizability of many of the studies due to small datasets lacking adequate participant diversity as well as methodological differences made it impossible to describe a single leading method. Further, the reliance on full recordings for segment imputation in some methods could prove prohibitive to real-time imputation. Future study is required to rectify these limitations and to properly investigate computational latency and requirements. **Significance:** This work provides novel insights into existing methods for EEG gap imputation, as it identifies current shortcomings in the literature and paves a way for a more generalizable solution to be achieved through future work.

## 1. Introduction

Electroencephalogram (EEG) is an electrophysiological tool broadly used across diagnostic, research, and clinical contexts that measures the electrical activity of the cerebral cortex [1,2,3]. EEG has long played a critical role in the diagnosis of neurological disorders, such as epilepsy, through the detection of pathological cerebral electrical activity and the elucidation of associated aberrant activity [2,3,4,5]. EEG-based research is critical in ameliorating insights into neurophysiological pathologies, as well as in the development of pharmacological interventions and therapies for seizures [3]. Additionally, EEG can also provide insights into cerebral processes underlying different cognitive phenotypes such as perception, attention, and memory [6]. EEG measurement is an integral element of intensive care monitoring due to the propensity of critically ill patients to experience seizures [7,8,9,10]. Patients are exposed to an increased risk of seizures as a secondary injury pattern from cerebral lesions, traumatic brain injury, infections, and substance abuse, amongst other pathologies [11,12]. Further, EEG has also been proposed as a modality to monitor the depth of sedation of comatose patients or during surgical procedures, helping to detect potential complications during anesthesia titration [1,13,14]. EEG signals are acquired non-invasively by electrodes placed on the surface of the scalp that detect and amplify the small electrical activity in the neurons of the underlying cortex [2,15,16]. In some instances, such as instances where seizures arise deep in the brain or where the exact epileptogenic zone must be determined with high spatial resolution, it is necessary to conduct invasive EEG (iEEG) using surgically implanted electrodes directly on the cortex or embedded inside cerebral tissue [17].

A pervasive issue in EEG measurement is the prevalence of artifacts that reduce the ability of these signals to accurately describe the underlying electrophysiological activity [18,19,20,21]. The presence of these non-viable signal segments can result in the misinterpretation of the signal waveform during analysis [1], potentially leading to inaccurate or missed diagnoses in a clinical context [22,23]. The small electrical potentials generated by the underlying cortical neurons measured during EEG recordings necessitate very high sensitivity. As such, signals are easily contaminated by noise and artifacts from a variety of physiological and environmental sources [21,24,25]. Physiological artifacts are produced by the electrical potentials generated by ocular, myogenic, and cardiac activity [1,18,21,24,25]. Environmental artifacts result from electrical interference, electrode displacement, high electrode impedance, patient movement, and patient sweating, amongst a litany of other sources [1,21,24,25]. While methodological techniques can be applied during acquisition to reduce the incidence of artifacts [18,21,24], the post-acquisition processing of EEG signals is integral to reduce or remove artifacts prior to improving the accuracy of the proceeding analysis.

A manual visual inspection of EEG data streams is the most rudimentary method of identifying artifacts, which is incredibly tedious and time consuming, introduces subjective bias into the annotated data, and is prone to human error [18,24]. As such, there has been a shift towards the semi- and fully automated identification and removal of artifacts using an algorithmic approach relying on various signal analysis methods. Methodologies have been developed to identify artifacts, with some capable of removing artifacts while maintaining the underlying signal. Existing methods rely upon signal processing techniques such as wavelet analysis, independent component analysis, and principal component analysis, amongst other signal processing and machine learning modalities for artifact identification and removal; these methods are discussed in detail in previously published reviews [18,26,27].

**Figure 1:**
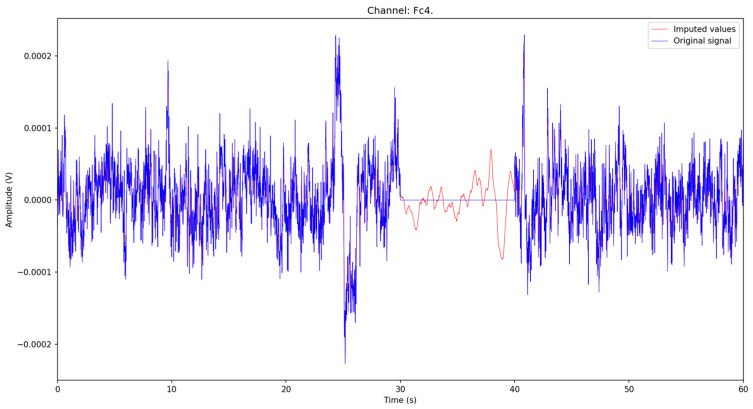
Example of EEG signal data gap imputation using the publicly available PhysioNet EEG Motor Movement/Imagery Dataset [28,29].

Following the identification of EEG signal artifacts, it is integral to maintain a continuous signal that accurately depicts the underlying electrophysiology. This necessitates highly accurate artifact removal or imputation methods for EEG signals. There is an expansive body of literature that discusses methods for artifact removal in EEG signals; however, these articles focus on techniques to remove artifacts that correspond to movement or contamination from other physiologic processes such as myogenic or cardiac activity. These methods remove artifacts from the EEG signal while maintaining the underlying EEG signal. There has been little investigation into methodologies that are able to interpolate EEG signals in instances where entire segments of data are ‘missing’ and there is no underlying signal that can be relied upon. These specific instances are common in equipment failures, resulting from instances of EEG sensors falling off or becoming disconnected. An example of such a data gap in an EEG signal being imputed is depicted in Figure 1 using EEG data from the publicly available dataset.

This review seeks to describe and evaluate the methods that have been developed thus far for the imputation or reconstruction of missing gaps in EEG signals that have been validated using experimentally recorded signals. A secondary objective is the potential elucidation of a leading method for this application. There currently exists no comprehensive investigation of existing EEG imputation methods for missing segments that have been validated using true EEG signals despite imputation being a critical next step proceeding EEG artifact identification. As such, this review serves as a foundational contribution to artifact management for EEG signals.

## 2. Methodology

This manuscript details a systematic scoping review that was conducted following the methodology outlined in the Cochrane Handbook for Systematic Reviews. This review and the reporting of the results were conducted following the Preferred Reporting Items for Systematic Reviews and Meta-Analysis (PRISMA) guidelines with the PRISMA Extension for Scoping Review [30,31]. The complete PRISMA checklist can be found in Supplementary File S1.

### 2.1. Ethical Considerations

The articles included and examined as part of this review were from previously published journals. As such, they are expected to have been vetted by these journals. This made it unnecessary to conduct any specific ethics approval review.

### 2.2. Search Question and Inclusion/Exclusion Criteria

The main question examined within this review was, “What imputation techniques exist for EEG signals that are able to reconstruct missing data?” Additional secondary questions focused on the effectiveness of each of the techniques included in the manuscripts reviewed, how each proposed method worked for interpolating EEG missing values, and the context for which they were designed. The articles included in this review were required to present an imputation methodology capable of imputing EEG signals where no underlying signal was available. The data used needed to have been collected from healthy or diseased human populations and possess a ‘ground truth’ signal to be able to quantify imputation quality. Articles that were excluded were non-English, review articles, those without a quantification of the performance of imputation methods, those interpolating entire channels, and those conducting imputation on EEG signals that were reliant on an underlying EEG signal within an artifact. Additionally, articles which presented algorithms that had been trained/tested/validated solely on simulated EEG signals, synthetic EEG without real EEG signals, or not involving empirical comparison to baseline methods were not included, as they do not represent true EEG signals for imputation. Signals possessing a ‘ground truth’ definitionally included masked EEG signals. These data streams needed to have values removed/missing to evaluate the performance of these methods in maintaining a continuous data stream in use cases where EEG signals were absent altogether or corrupted to the severity that no underlying signal could be salvaged. Further, included methods could not be reliant on inter-electrode imputation, as this would limit the utility of such methods in instances of single-channel recordings or corrupted/missing segments that span several electrodes. The included articles were grouped based on types of imputation methods. For each article within each group, EEG imputation and artifact removal studies were included if they explicitly implemented or evaluated a quantitative method for recovering missing or artifactual EEG data and provided at least one quantitative metric.

### 2.3. Search Strategy

Searches were conducted across five databases, which included BIOSIS, SCOPUS, EMBASE, PubMed, and Cochrane Library. The initial search was conducted on 24 September 2024, with the search results covering the entire period from the inception of each database up to the search date. The search was then updated on 16 June 2025. Based on the filtering limitations of each of these databases, the second search of EMBASE and SCOPUS had the results covering the period, from the beginning of 2024 to 16 June 2025. The second search of PubMed, Cochrane Library, and BIOSIS had the results covering 24 September 2024 to 16 June 2025. A tailored search string was conducted to encapsulate imputation for all cerebral bio-signals (both pressure-flow signals and electrophysiologic signals), provided in Supplementary File S2. The results from each of the five databases were combined and de-duplicated. This provided an extensive and comprehensive list of all unique articles pertaining to the search string.

### 2.4. Study Selection

The unique articles that were identified across the search conducted across the five databases were then systematically reviewed following a two-stage, two-reviewer approach. For this first stage, two reviewers independently screened the titles and abstracts of the articles and compared them to the inclusion/exclusion criteria. The studies that were included after the first stage were then screened using the full text, again comparing them to the inclusion/exclusion criteria. Any disagreements after either of these stages were resolved by a third party. The original search’s first and second screenings were conducted by TB and IM, with disagreements resolved by FAZ. The updated search’s first and second screenings were conducted by XNS and JR, with disagreements resolved by TB and FAZ. Initially, the search string for this review encapsulated gap imputation methods for all cerebral physiological signals. The initial search had a volume of articles that needed to be included that could not be synthesized and analyzed in adequate detail in a single manuscript. As such, the screening results were bifurcated into two manuscripts (missing gap imputation in cerebral electrophysiological signals and missing gap imputation in cerebral pressure-flow signals). Following the initial search, a priori screening was used to solely encapsulate electrophysiological signal missing data gap imputation. The initial search was designed to encapsulate all cerebral bio-signal imputation methods, and the screening of the initial search kept any method designed to impute missing values for any cerebral bio-signals. As such, during a priori screening to include only cerebral electrophysiological signals, selection bias was able to be limited, as the initial search and screening was exhaustive. As such, articles categorized as ‘full-length articles meeting inclusion criteria’ solely detailed gap imputation methods for cerebral electrophysiological signals, with methods developed for cerebral pressure-flow signals being omitted on the basis of ‘wrong signal type’.

There were a portion of the methods identified in this review that were presented as ‘artifact removal’ or ‘artifact interpolation’ methods. Definitionally, these terms may typically signify signal denoising or interpolation using an underlying signal; however, a granular analysis of the structure of the methods confirmed that the methods included did not utilize any portion of the underlying signal in the constructed/imputed signal. As such, these methods were justifiably included in this review despite their titles.

### 2.5. Data Collection

Data collection was conducted by YS, MM, and TB on the final list of articles for this systematic review. The extracted data used for the analysis in this review is presented in Supplementary File S3 in the form of structured tables. Data extracted from each article (if information was available) are organized as follows:

1. Reference—author name and citation;
2. Subject information—number of subjects, duration of recording and number of trials/sessions, external stimuli applied during recording, and dataset availability;
3. Signal type and duration—type of signal recorded, sampling rate used, and any preliminary filtering applied (e.g., down sampling);
4. Missing data characteristics—methodology used to induce gaps in the data, proportion of the data that was ‘missing’;
5. Location and number of sensors—number of sensors used for data collection and anatomical location of placement;
6. Imputation technique used—detailed description of the imputation methodologies presented as the focus of the article;
7. Methods compared—any other methods used to compare performance to the imputation methodologies presented;
8. Effectiveness—quantitative data describing the effectiveness of the proposed methods of imputing the EEG signal;
9. Results of the study—major results of the article, specifically focusing on the relative performance of imputation methods;
10. Limitations—any limitations identified in the study methodology, imputation methodology, or feasibility of implementation of the methodology, amongst other identified limitations.

The data extracted from each article in these tables enabled the critical analysis and comparison of the article included in this review.

### 2.6. Assessment of Bias

All articles included have been published in academic journals; as such, biases were assumed to have been screened as part of their independent peer-review associated with the respective journals. However, no formal risk-of-bias assessment was conducted as part of this scoping review.

## 3. Results

**Figure 2:**
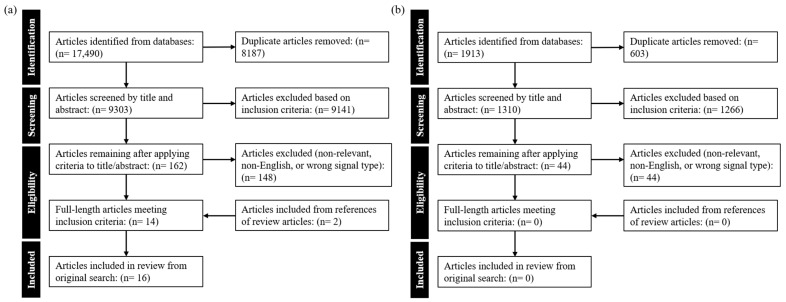
PRISMA flowchart for systematically conducted scoping review from searches on (**a**) 24 September 2024 and updated on (**b**) 16 June 2025.

The results of the search across BIOSIS, SCOPUS, EMBASE, PubMed, and Cochrane Library databases yielded 17,490 results. The de-duplication process revealed that there were 9303 unique articles to be included in this review. Following the first screening phase, 162 articles were deemed eligible based on inclusion/exclusion criteria. The number of articles that were deemed eligible based on full-length screening was 14. An additional search through the reference sections of the included articles led to the inclusion of two more articles. The initial updated search yielded 1913 results. The de-duplication process conducted revealed that there were 1310 unique articles included in this review. Following the first screening phase, there were 44 articles deemed eligible based on inclusion/exclusion criteria. The number of articles that were deemed eligible based on full-length screening was zero. An additional search through the reference sections of some of the considered articles yielded no other articles. As such, there are a total of 16 published works that present imputation methods that quantifiably address missing data gaps or artifactual EEG signals included in this review. These are depicted in a PRISMA flow diagram in Figure 2a,b.

**Figure 3:**
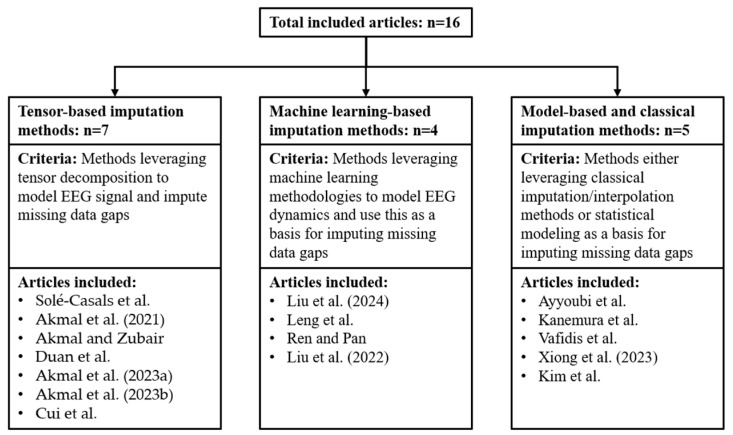
Categorization of articles based on methodology architecture [32,33,34,35,36,37,38,39,40,41,42,43,44,45,46,47].

The articles that presented methods for data gap imputation in EEG signals were sorted into three categories based on the architecture of the methods used. The categories were (i) tensor-based imputation methods, (ii) machine learning-based imputation methods, and (iii) model-based and classical imputation methods. The categorization of these articles is presented in Figure 3.

### 3.1. Tensor-Based Imputation Methods

Since raw EEG data can generally be expressed as a multidimensional array with three dimensions (time x channels x trials) [38], tensor factorization methods seek to interpolate missing data through a decomposition of such *n*-dimensional data streams into dynamic characteristics (or filters) latent within valid signal segments. The review criteria resulted in seven articles that were capable of imputing missing EEG signals using a tensor-based methodology [32,33,34,35,36,37,38]. One of the seven articles was designed to impute noisy segments; however, the underlying valid signal in the noisy segments was not used in the imputation algorithm, as noisy segments were completely discarded before tensor-based imputation [32]. The other six were specifically designed for the imputation of missing segments [33,34,35,36,37,38]. As such, all identified algorithms can be applied for the imputation of missing EEG data gaps. Generally, the tensor-based methodologies were optimized using existing data and were used to fill missing data segments. A summary of the relevant data extracted from these articles is depicted in Table 1. Included in this table is pertinent study information, including the presence of pathophysiology for study participants, age demographic information, the number of electrodes used for data collection, and missing data characteristics. Also included in this table is a summary of the algorithm, its quantified effectiveness, training/testing required, and computational time. More information regarding the study methodology, imputation algorithms, quantitative effectiveness, and results of these studies are included in Supplementary File S3 (Table S2).

Across the seven studies categorized as tensor-based methods, all seven studies that used presumed healthy participants (no disease stated) [32,33,34,35,36,37,38]. The median age of the participants included calculated between studies was 25 (IQR: 22 to 28) [34,35], with five studies not providing age demographic information [32,33,36,37,38]. The mean number of electrodes used was 4 (IQR: 3 to 6), with sampling rates ranging from 128 to 256 Hz.

Canonical Polyadic (CP) decomposition (CPD) is the basis of several of the methodologies included in this work. CPD involves the iterative optimization of an objective function that minimizes the difference between tensors constructed using actual EEG data and reconstructed tensors for missing data [34]. However, due to a standard assumption that the data is complete, missing data segments often lead to suboptimal convergence. CP is evaluated in three of the articles included in this review [33,34,36]. CP Factorization with Weighted Optimization (CP-WOPT) builds on this concept by optimizing a weighted objective function that ignores missing datapoints when constructing the tensor, theoretically improving on a shortcoming of the CPD method, as it uses the entire dataset for tensor construction [34]. The CP-WOPT method was presented in several articles and was used as a basis for comparison for several other tensor-based methods [32,33,34,35,36,37]. The CPD and CP-WOPT algorithms serve as the most basic approaches to tensor completion. Solé-Casals et al. [32] expanded on this concept, proposing the use of more nuanced tensor completion methods. Namely, the authors presented the 3D Patch-based Tensor Completion (3DPB-TC) algorithm, which breaks down large tensors into smaller overlapping cubes (patches) and derives sparce representations capable of capturing local dynamics. To address output instability caused by manual rank selection, the authors introduce a Bayesian CP Factorization (BCPF) method. Rather than treating rank as a fixed parameter, BCPF applies sparsity-inducing priors to factor matrices. These priors are then tuned through Bayesian inference for automated tensor rank selection. Additionally, the authors introduced a High-Accuracy Low-Rank Tensor Completion (HaLRTC) method. This method relies on the assumption that clean EEG data is highly correlated (low-rank), whereas noise adds unstructured complexity (high-rank). HaLRTC thereby reconstructs missing data segments by minimizing the generalized trace norm of a tensor, effectively recovering clean signal portions and filtering out high-rank noise dynamics [32]. Duan et al. [35] not only compared CP-WOPT, BCPF, and HaLRTC algorithms but also presented the Simultaneous Tensor Decomposition and Completion (STDC) algorithm, which is a Tucker decomposition-based tensor completion method that relies upon maximum a posteriori estimation for the resultant tensor. The final tensor-based method is the Xavier-initialized Weighted CP (X-WCP) algorithm, which aims to improve upon the CP-WOPT algorithm by using Xavier initialization for weight selection to speed up convergence time [36].

The CP-WOPT algorithm was evaluated against the CPD algorithm in multiple articles [33,34,36]. These methods were compared to the Non-Negative Matrix Factorization (NMF) algorithm, with the performance being quantified using root mean error (RME). In each of these three articles, the CP-WOPT method had the strongest performance, even at 50% missing data, where the RME values for NMF, CPD, and CP-WOPT were 0.61, 0.76, and 0.16, respectively, for one of the datasets [36]. Further, less missing data seems to result in better reconstruction accuracy. Using the previously mentioned dataset, the RMSE values at 20% missing data for NMF, CPD, and CP-WOPT were 0.24, 0.61, and 0.07, respectively. The performance of the CP-WOPT algorithm compared to the CPD algorithm indicates that the use of tensor-based reconstruction methodologies is improved when only valid EEG data are utilized for tensor construction of missing segments. The computational time to process the testing dataset of 1152 × 3 × 40 timepoints with various amounts of missing data also indicated that the run-time of the CP-WOPT algorithm was fast, processing data with 50% missing in 6.7 s, but this was close to CPD and NMF at 5.1 and 4.0 s, respectively [36]. The BCPF method had strong performance, achieving a relative standard error result below 0.1 for missing data ratios of 70% [38]. The CP-WOPT algorithm indicated strong performance in several articles; however, the use of more nuanced tensor-based reconstruction algorithms seemed to further improve performance in certain comparative analyses. A robust comparison of tensor-based method performance was conducted by Solé-Casals et al. [32]: the performance of the CP-WOPT, 3DPB-TC, BCPF, and HaLRTC algorithms was evaluated. Across a range of missing samples, the NRMSE values for the reconstructed EEG data compared to the ‘ground truth’ was on the order of 10^−1^; however, the HaLRTC method had the strongest performance, achieving a normalized root mean square error (NRMSE) value of ~10^−2^ even at a missing data rate of 20% [32]. However, HaLRTC was outperformed by the STDC algorithm in the work by Duan et al. [35], which indicated that the negative logarithm NRMSE (LNRMSE) was lower for this method across variations in both number of time series affected by missing data and the duration of the missing segments themselves [35]. The use of Xavier initialization also had superior performance compared to CP-WOPT, achieving an RME value of 0.15 compared to the 0.18 of CP-WOPT for 50% missing data, and was also was faster, converging in 51.59% less time [36]. The computational hardware required to conduct imputation using these methods was generally under reported. Three of these articles reported computational specifications for the units used. Akmal et al. (2021) [34] and Akmal and Zubair [33] reported the use of relatively simplistic CPUs requiring only six GBs of RAM. Akmal et al. (2023b) [37] reported the use of more complex GPU processing with 256 GB of RAM.

### 3.2. Machine Learning and Deep Learning Imputation Methods

Machine learning and deep learning approaches have shown notable advancement in modeling complex non-linear relationships within EEG data for artifact correction and missing segment reconstruction. There were four articles that presented machine learning-based EEG reconstruction [39,40,41,42]. Two of these articles clearly presented methods developed for imputing missing values [39,40]. One of the articles presented a method as being able to impute noisy EEG segments; however, further examination revealed that this method treated the identified noisy segments as ‘missing’ before conducting imputation and was specifically tested on missing data gaps [41]. One of the articles indicates that the method developed was used for imputing ‘artifacts’; however, further examination showed that the ‘artifacts’ being imputed were segments that had been set to NaN values [42]. As such, all of the articles presented methods capable of imputing missing data segments in EEG data streams. A summary of the relevant data extracted from these articles is depicted in Table 2. Included in this table is pertinent study information, including the presence of pathophysiology for study participants, age demographic information, the number of electrodes used for data collection, and missing data characteristics. Also included in this table is a summary of the algorithm, its quantified effectiveness, training/testing required, and computational time. More information regarding the study methodology, imputation algorithms, quantitative effectiveness, and results of these studies are included in Supplementary File S3 (Table S3).

Across the four studies categorized as machine/deep learning methods, one of them was applied to an epileptic patient cohort [39], while the remaining three were applied to presumed healthy individuals (no disease stated) [40,41,42]. Two of the studies did not provide information regarding EEG sampling rate [39,40], one study used data recorded at 250 Hz [41], and one study used three different datasets all down sampled to 128 Hz [42]. Two studies provided information regarding the number of electrodes used: Leng et al. [40] used 14 electrodes and Ren and Pan [41] used 22 electrodes placed according to the 10–20 system, which is a standardized methodology for electrode placement [48]. Liu et al. (2024) [39] did not report the number of electrodes used. Liu et al. (2024) [39] did report the number of participants from which data was collected (*n* = 10). Liu et al. (2022) reported the number of participants in each of the three datasets (*n* = 12, 26, and 9, respectively). The first of these three datasets was EEG data recorded from subjects exposed to stimuli at varying rates while on a stationary biking; the dataset was split into two sub-groups based on whether the participant was pedaling or not [49]. The second of the two datasets was EEG data recorded from individuals performing a ‘P300 Speller’ task [50]. The third dataset was EEG data recorded from individuals conducting motor imagery tasks [51]. The remaining two articles did not report the number of participants [40,41].

Each included article presented a machine learning-based method to impute missing data from EEG data; their results were presented within their respective experimental scopes, independent of comparative benchmarks. Thematically, the reviewed methods leveraged inherent time/frequency-domain dynamics and/or correlative structures present in EEG data to model normal signal behavior and impute missing segments. Liu et al. (2024) [39] presented the Bi-directional Long Short-Term Memory Network Extreme Learning Machine-Elastic Net Self-Attention Inception Time Network (BiLSTMELM-ENSAIT) method, which was able to capture future, past, and long-term dependencies of EEG data streams using the Bi-directional Long Short-Term Memory Network (BiLSTM) layer. The training was accelerated and overfitting was minimized using the Extreme Learning Machine (ELM) layer [39]. Leng et al. [40] presented the Spectral Clustering Augmentation Pseudo-label Conditional Generation Adversarial Imputation Network (SCAPC-GAIN). This method was constructed based on a Generative Adversarial Network, which typically involves training two components: a generative model to understand the underlying data and a discriminative model for data classification [52]. The SCAPC-GAIN model construction involved two steps for training (pre-training and formal training phases) [40]. In the pre-training phase, the authors generated initial estimates for missing data and then conducted spectral clustering to generate pseudo-labels. Missingness augmentation was used to improve the generator using an auxiliary classifier. The formal training phase was used to guide the model’s construction. A 5-fold cross-validation was conducted to evaluate the model efficacy [40]. Ren and Pan [41] introduced the Time–Frequency Cross-Mutual Information-Convolutional Neural Network-Long Short-Term Memory (TFCMI-CNN-LSTM) model. This model leverages wavelet transformation to obtain time–frequency power information from each channel and uses this to determine linear and non-linear correlations between channels. By organizing them by similarity, this method can leverage correlations and mutual information to determine time–frequency mutual information values. Previous time-series data from the channel with missing information, as well as that of channels indicated to be of significance via Time–Frequency Cross-Mutual Information (TFMCI), are used by a Long Short-Term Memory Convolutional Neural Network (LSTM-CNN) to predict missing data [41]. Liu et al. (2022) [42] also leveraged a BiLSTM architecture in the proposed State-Based Recurrent Imputation for EEG (SRI-EEG) algorithm. SRI-EEG incorporated an input mask to only use valid data within the model, also considering gap duration, spatial correlations of adjacent channels, and the ‘state’ of trials as additional features in the model [42].

Each of these presented methods showed strong effectiveness in reconstructing missing EEG data relative to ‘ground truth’ signals. The findings of reviewed methods indicated variable efficacies dependent on model architecture and missing data rates. For instance, Liu et al. (2024) [39] indicated that the BiLSTMELM-ENSAIT algorithm outperformed an Imputation-LSTM (Im-LSTM) model during missing data reconstruction at various levels of structured missing data rates (10%, 20%, 30%, and 50% of total data). At a missing data rate of 50%, BiLSTMELM-ENSAIT achieved an RMSE of 0.234 and an R^2^ value of 0.723, compared to an RMSE of 0.248 and an R^2^ value of 0.712 for the Im-LSTM model. The exception to this was, at a missing data rate of 20%, the BiLSTMELM-ENSAIT achieved an RMSE of 0.168 and R^2^ of 0.826, while the Im-BiLSTM had slightly better results, with an RMSE of 0.160 and R^2^ of 0.822. Leng et al. [40] indicated superior performance for the SCAPC-GAIN algorithm across datasets with missing data rates of 20% to 80% (step size of 10%) compared to conceptually similar methods relying on conditionally generative architectures relying on pseudo-labels. At a missing data rate of 20%, the SCAPC-GAIN algorithm achieved an RMSE value of 0.124 compared to the Generative Adversarial Imputation Network (GAIN), Pseudo-label Conditional GAIN (PC-GAIN), and Missingness Augmentation GAIN (MA-GAIN) algorithms, which achieved RMSE values of 0.142, 0.2107, and 0.154, respectively, at that missing data rate [40]. While Ren and Pan [41] did not perform comparative benchmarking with their TFCMI-CNN-LSTM model, they investigated the use of correlated channels as a feature input for the imputation model in data reconstruction. In a dataset where segments of 500 milliseconds were treated as missing, features in time–frequency cross-channel correlations produced the strongest performance compared to the use of a random channel as a feature or using no other channel as a feature. Using correlated channels as features, the TFCMI-CNN-LSTM algorithm achieved RMSE, mean absolute error (MAE), and Spearman Rank results of 3.512, 2.787, and 0.828, respectively [41]. This model is indicated as being ‘more suitable’ for real-time applications; however, there is no quantification to support this claim. Liu et al. (2022) [42] indicated superior performance to other model-based or machine learning-based methods, achieving the lowest results in MAE and RMSE across all three datasets. At a 10% missing data rate, the SRI-EEG method achieved RMSE values ± standard deviation of 88.192 ± 1.70 for dataset 1a, 12.142 ± 0.45 for dataset 1b, 69.985 ± 0.85 for dataset 2, and 0.889 ± 0.01 for dataset 3 (dataset 1 was split into ‘noisy’ (1a) and ‘denoised’ (1b) based on task being completed while pedaling a stationary bike or not). In contrast to the next-best methods, which were the Bidirectional Recurrent Imputation for Time Series (BRITS) and Multi-Directional Recurrent Neural Networks (MRNNs). BRITS achieved RMSE results of 116.477 ± 2.12 for dataset 1a, 13.474 ± 0.51 for dataset 1b, 70.321 ± 1.13 for dataset 2, and 0.911 ± 0.04 for dataset 3. MRNN achieved RMSE results of 93.212 ± 1.86 for dataset 1a, 13.021 ± 0.62 for dataset 1b, 72.594 ± 0.72 for dataset 2, and 0.925 ± 0.03 for dataset 3, indicating a significant difference between datasets, potentially attributed to the tasks being performed affecting the signal dynamics or introducing noise. Only a single method included in this section reported the computer hardware specifications used for imputation: Liu et al. (2024) [39] used a unit with CPU and GPU processing capacity with 64 GB of RAM.

### 3.3. Model-Based and Classical Imputation Methods

The model-based and classical imputation methods category encapsulates non-deep learning methodologies capable of EEG missing data reconstruction. These methods rely on dynamic modeling or local statistical modeling to estimate missing data segments [43,44,45,46,47]. Three of these articles presented methods specifically designed to impute missing values [43,44,47]. Two of the included methods for the ‘artifact removal’ of TMS-related artifacts; however, further examination revealed that these methods were effectively treating the TMS artifacts as ‘missing’ before imputation [45,46]. A summary of the relevant data extracted from these articles is depicted in Table 3. Included in this table is pertinent study information, including the presence of pathophysiology for study participants, age demographic information, the number of electrodes used for data collection, and missing data characteristics. Also included in this table is a summary of the algorithm, its quantified effectiveness, training/testing required, and computational time. More information regarding the study methodology, imputation algorithms, quantitative effectiveness, and results of these studies are included in Supplementary File S3 (Table S4).

Five articles were identified as presenting model-based and classical imputation methods [43,44,45,46,47]. Two of these studies used data collected from an epileptic population [43,45], two studies used data collected from healthy or unspecified populations [44,46], and one study used both an epileptic population and unspecified populations [47]. Ayyoubi et al. [43] conducted imputation on intracranial EEG (iEEG) data. Additionally, Vafidis et al. [45] and Xiong et al. [46] used data collected using a transcranial magnetic stimulation EEG (TMS-EEG) system. The remaining two articles used typical EEG data [44,47]. Four of the articles reported the number of participants, which were three for Ayyoubi et al. [43], nine for Kanemura et al. [44], one for Vafidis et al. [45], and twelve for Kim et al. [47]. Xiong et al. [46] did not report the number of participants used. There was also a significant range in the number of electrodes used for data recording. Ayyoubi et al. [43] recorded data using a clinical system with the capacity to record 256 channels; however, this study was focused on comparing clinical systems to a wireless system, as such channels were limited to 32 to align with that system. Kanemura et al. [44] and Vafidis et al. [45] used systems with 22 and 60 electrodes, respectively. Xiong et al. [46] used data collected with 64 electrodes [46]. Kim et al. [47] used data from three different datasets (from Universitäts Klinikum Bonn [53], Event Related Potential [54], and BCI Completion II [55]); these contained data collected using 100, 31, and 64 channels, respectively [47].

Each article presented one or more methodologies for interpolating missing data for EEG signals. Ayyoubi et al. [43] evaluated three classical imputation methods for their ability to reconstruct ‘packet loss’ from clinical iEEG devices. These evaluated methods included previous value replacement (PVR), linear interpolation (LI), and spline interpolation (SI). There is no in-depth description of these algorithms. PVR indicates the use of the last valid timepoint before the missing data gap to replace each of the missing timepoints. LI and SI describe fitting linear and low-order polynomials to data immediately before a gap and using these functions to interpolate missing data. However, it was not clear from the article as to how many points were used to model the signal prior to the gap.

Kanemura et al. [44] proposed statistical model-based multi-variate autoregressive (MAR) imputation. At a high level, this method autoregressively computes missing values by modeling multi-channel dynamics in past and present data. This method is compared to classical imputation methods, including nearest-neighbor interpolation (NNI) and linear interpolation. Vafidis et al. [45] proposed a more complex model that they refer to as a ‘gap filling method’. This method identifies viable signal segments that maximally correlate with the signal patterns present at gap boundaries and models the local state space to interpolate missing data segments [45]. Vafidis et al. [45] were investigating the efficacy of the model-based gap-filling and shape-preserving piecewise cubic spline interpolation methods, as well as a FastICA- and principal component analysis (PCA)-based algorithms, in removing and interpolating transcranial magnetic stimulation (TMS) artifacts in EEG signals. The gap-filling and shape-preserving piecewise cubic spline interpolation methods disregarded artifacts for the reconstruction task, hence their consideration in this review given the inclusion/exclusion criteria. The FastICA- and PCA-based methods relied on the underlying EEG for imputation and thus did not satisfy inclusion criteria. Xiong et al. [46] proposed the 3D Adaptive Rational Quadratic Hermite Interpolation (3DARQHI) method and compared it to several imputation algorithms, including the gap-filling, Piecewise Cubic Hermite Interpolation (PCHI), and Structured Output Unsupervised Denoising (SOUND) algorithms. The 3DARQHI algorithm mathematically reconstructs missing EEG signals using rational quadratic Hermite interpolation [56], but specifically optimizes the shape parameter to accommodate the non-linearity of EEG signals. Kim et al. [47] proposed a PCA-based method that uses expectation maximization (EM) to refine predictions for missing EEG segments and is referred to as the Incremental Expectation Maximization Principal Component Analysis (iEMPCA) method.

Ayyoubi et al. [43] investigated the ability of different imputation methods to recover EEG signals that had been synthetically removed at a rate of 10%. Power spectral density (PSD) was used to evaluate the efficacy of the interpolated signals compared to the ‘ground truth’ signal. LI and SI reported superior performance to PVR within this study. Graphs of PSD indicated that the performance of these methods deteriorated in their recovery of higher-frequency components as defined by PSD values. However, LI and PI still indicated strong performance, with PSD values close to that of the raw data at 500 Hz, with an approximate difference of 2 to 3 dB. Kanemura et al. [44] introduced gaps of 0.25 s into EEG data in 5 of the 22 channels being recorded. The MAR algorithm had the best performance in reconstructing these gaps, achieving a mean RMSE value of 2.143 across all trials and outperforming NNI and LI, which had mean RMSE values of 3.407 and 19.333, respectively [44]. Vafidis et al. [45] introduced TMS artifacts (treated as missing by included methods) at three different portions of the EEG recordings corresponding to specific physiologic events (resting EEG, beginning of epileptiform discharge, and during epileptiform discharge). There were differences between the performances of imputation methods depending on which portion of the data was investigated. At resting EEG and at the beginning of epileptiform discharge, gap-filling achieved RMSE values of 13.95 and 20.23 for each respective physiological event, compared to the 13.72 and 20.54 achieved by the shape-preserving piecewise cubic spline interpolation method. During epileptiform discharges, the gap-filling method performed poorer with an RMSE value of 44.29 compared to the 18.23 achieved by the shape-preserving piecewise cubic spline interpolation method. This highlights the constraints of the gap-filling method in modeling non-stationary and heteroscedastic EEG data (i.e., heterogenous dynamic means and variances during rest and seizure activity). Xiong et al. [46] induced TMS artifacts in EEG data where artifacts were treated as missing data; the 3DARCHI outperformed all methods against which it was compared by SNR, RMSE, and MAE to the ‘ground truth’ signal within the Xiong et al. work [46]. The 3DARCHI algorithm achieved RMSE values of 0.34. Other methods that were outperformed by the 3DARQHI algorithm included the gap-filling and SOUND algorithms, which achieved RMSE values of 19.74 and 4.64 respectively. Kim et al. [47] compared the iEMPCA algorithm to two other decomposition-based algorithms: Robust Expectation Maximization Principal Component Analysis (Robust EMPCA) [57] and Missing Value Singular Value Decomposition (MSVD) [58]. The performance of these algorithms were quantitatively compared using RMSE for their ability to interpolate missing data segments randomly initiated at rates of 5%, 10%, and 15% for each of the three datasets [47]. Average RMSE values were calculated across the missing data segment rates for each dataset [47]. The iEMPCA algorithm had the strongest performance for the Universitäts Klinikum Bonn [53] and Event-Related Potential [54] datasets, achieving RMSE values of 0.0344 and 0.0163, compared to the 0.0431 and 0.0220 achieved by the Robust EMPCA algorithm and the 0.0378 and 0.0182 achieved by the MSVD algorithm [47]. For the BCI Completion II dataset [55], the iEMPCA algorithm underperformed, achieving an RMSE value of 0.0313, in contrast to the Robust EMPCA and MSVD, achieving RMSE values of 0.0300 and 0.0262, respectively.

Xiong et al. [46] presented the computational time required to execute the imputation for three periods (1200 timepoints) of 64-channel EEG data, which was the fastest for the 3DARQHI algorithm at 1.45 s using an HP Z840 with a 16-core Intel Xeon E5-2620 CPU processor at 32 GB of memory. In comparison, PCHI ran for 2.25 s, SOUND for 3.54 s, and gap-filling for 5.86 s. The Xiong et al. [46] article was the only one to report computational hardware specifications for the analysis conducted. The remaining articles included in this section did not provide information regarding computational time or performance for real-time utility. Kim et al. [47] indicated that their developed article was designed for real-time applicability, but they did not provide estimates or a quantification of computation time.

## 4. Discussion

EEG is a broadly used clinical tool used to record electrophysiological activity in the brain with high temporal resolution. These signals are recorded invasively and non-invasively in varying environments across healthcare. EEG signals are critical in the diagnosis of neurological disorders, understanding the brain’s response to different pharmacological treatments, and in providing insights into cerebral processes. However, signal artifacts are very common in EEG recordings, which degrade the signal quality, utility, and accuracy of diagnoses. A subsection of EEG signal artifacts are segments where any EEG activity is completely absent because of sensor disconnection or failure. These artifacts are particularly difficult to interpolate due to the lack of underlying EEG signal that can be used as a basis for signal reconstruction. This review sought to identify methodologies that have been developed to interpolate completely missing segments of EEG data that have been validated using true ‘ground truth’ EEG signals. Across the initial and updated search, there were a total of 16 articles that were included in this review, as they satisfied the established inclusion/exclusion criteria. However, there were several articles initially included that needed to be removed on the basis of these criteria.

The primary criterion for inclusion in this review was the presence of a ‘ground truth’ signal. There were several articles that presented imputation methods that were tested on artifact-ridden EEG signals. The algorithms were applied to interpolate the missing or artifactual data, but, without a ‘ground truth’, it was impossible to describe how well the signal was being reconstructed. The included articles needed to possess this ‘ground truth’ to properly quantify imputation quality, which led to several articles being excluded [59,60,61,62,63,64,65,66,67,68,69,70]. Included articles also needed to quantify the fidelity of the interpolated signals relative to this ‘ground truth’. The most common of such metrics were RMSE, MAE, and correlation scores. However, several identified articles did not quantify the methods’ efficacy in reconstructing EEG signals, resulting in their exclusion from this review [71,72,73,74,75,76,77,78,79,80,81,82,83,84,85,86]. This review aimed to elucidate a generalizable solution for imputation in EEG signals where no underlying signal was available. As such, this led to the exclusion of several articles presenting methods that did not satisfy the intended use-case for this review. Several articles were excluded due to imputation of artifact-laden data with underlying EEG signals [84,87,88,89,90,91,92,93,94,95,96,97,98,99,100,101]. Examples of such studies included those that interpolated EEG signals in the presence of electromyogram (EMG), electrooculogram (EOG), and electrocardiogram (ECG), or motion-based artifacts added to the signals. In such examples, there remains underlying EEG waveform information that the methods presented in these articles aim to salvage using different methodologies. This makes these articles out of the scope of this particular review, as this review focuses on the imputation of gaps where there is no underlying EEG signal whatsoever, as is the case in sensor disconnections. Additionally, the focus of this review was on gap imputation rather than entire missing channels. As a result, articles presenting methods for full-channel imputation were excluded [102,103,104,105,106,107]. This review aimed to discuss articles presenting methods that could impute segments of missing data; as such, methods that imputed entire missing channels would have required discarding entire channels of EEG data in instances where segments were missing to facilitate imputation. Furthermore, one article was excluded from this review because it solely interpolated single missing datapoints, which did not describe true gaps, but rather interpolation [108]. One additional article was also excluded despite conducting an imputation of EEG signal because, despite conducting imputation on EEG, the results quantified the effectiveness of the imputation of EEG combined with other vital signals [109]. Methods that were exclusively tested on gaps in synthetic EEG data were not considered in this review as they do not assuredly describe the morphology of real EEG signals. The focus of this review was to present methods developed and tested for the imputation of missing data gaps in real EEG signals capable of imputing signals in a context where no underlying signal could be leveraged.

Included studies in this review were organized by general methodological similarities into three categories: tensor-based imputation methods, machine learning- and deep learning-based imputation methods, and model-based and classical imputation methods. The CPD and CP-WOPT algorithms were the most rigorously studied of the tensor-based methodologies, exhibiting strong performance across several articles [32,33,34,35,36,37]. These methods served as the inspiration for the development of several more nuanced and complex tensor-based methods such as the HaLRTC, STDC, BCPF, and X-WCP algorithms [32,35,36,38], which improved on the performance of the CPD and CP-WOPT algorithms. However, these tensor-based algorithms were applied retrospectively to perform EEG imputations, allowing them access to full EEG recordings. Because EEG data streams are often being leveraged in clinical settings, real-time utility is a consideration for live-time prognosis and diagnosis. In its current form, the reliance on full datasets hinders their efficacy in real-time reconstruction tasks, where missing gaps arise unexpectedly. Alternatively, in certain diagnostic or research contexts, where entire datasets can be used and computational requirements and lag time are less impactful, complex tensor-based methods may be able to produce more accurate imputations. Unlike the machine learning and statistical modeling approaches explored in this review, the authors of tensor-based imputation methods conducted comparative benchmarking with varying methods, which helped to illustrate the nuances of how tensor-based methods garner superior imputation accuracy.

Across the four machine learning and deep learning imputation methods that were included in this review, there were some methodological similarities in the construction of the models. Liu et al. (2024) [39], Ren and Pan [41], and Liu et al. (2022) [42] presented methods leveraging the LSTM architecture to capture the long-term temporal dependences in EEG data. However, methodological comparisons proved difficult due to architectural heterogeneity among the various models. For instance, the BiLSTMELM-ENSAIT algorithm leverages the BiLSTM model designed to capture both the past and future temporal structures, as well as the long-term dependencies [39]. Despite also using an LSTM architecture, the TFCMI-CNN-LSTM algorithm specifically leverages time–frequency cross-mutual information to select correlated adjacent channels to serve as features for the CNN-LSTM model for imputation [41]. The SRI-EEG model differs from the two previously mentioned models, as it takes into account gap duration, spatial correlations, and the ‘state’ of the trial being interpolated as additional features [42]. Leng et al. [40] proposes the SCAPC-GAIN algorithm, relying on a GAN architecture. Their proposed model generates pseudo-labels using spectral clustering to train an auxiliary classifier during the pre-training phase and integrates the auxiliary classifier to constrain the generator in the GAN network to accurately interpolate missing EEG signals. Each of the machine learning and deep learning imputation algorithms were compared to different methodologies; however, there was no article that performed comparisons to the other included methodologies. Quantification regarding the computational requirements, computational latency, and potential for real-time applicability of these methods was absent across this section of the literature. Predominant concerns for more complex models, such as machine learning models, are that they often rely upon computationally heavy processing [108], which can be beyond the capacity of what is available in typical clinical settings. Additionally, the computational latency associated with this processing, can prohibit real-time utility, potentially resulting in the obsolescence of the data for prognosis or diagnosis. However, in the presence of clinical and diagnostic settings where computational hardware is more advanced, machine learning-based methods may be able to produce more accurate imputations without requiring access to entire datasets and with potentially minimal lag time.

Under the current inclusion/exclusion criteria, a wide variety of methods were categorized as model-based or classical imputation techniques. For instance, Ayyoubi et al. [43] presented relatively simplistic methods for imputation using either PVR, LI, or SI, while Kanemura et al. [44], Vafidis et al. [45], and Xiong et al. [46] proposed iterative algorithms that optimized the estimates of local temporal dynamics in EEG signals. Similar to the machine learning and deep learning methods, there was little intercomparison of the performances of these methods within any article included in this section. The exception to this was Xiong et al. [46], who indicated a superior performance of the 3DARQHI algorithm compared to the gap-filling method proposed by Vafidis et al. [45]. It was particularly difficult to compare the performance of the simple methods presented by Ayyoubi et al. [43] to the more complex models, as imputation was quantified using PSD as opposed to RMSE or like metrics used by the other methods [44,45,46]. There was no quantification or indication of any real-time utility or computational latency in many of these models. The reliance on the local state space modeling and estimate optimization of the more complex model-based methods may be prohibitive to real-time utility or garner significant computational lag, making the lack of any evaluation of computational latency and requirements a gap in this literature. The exception to this was Xiong et al. [46], who conducted a study on the computation lag time to process 1200 timepoints of TMS-EEG signals, achieving a mean runtime of 1.45 s for their proposed 3DARQHI algorithm. However, with the lack of sampling rate provided in this work, it was impossible to determine how close to real-time applicability this was. The classical imputation and model-based imputation methods are likely comparatively the most computationally ‘lightweight’ methods, giving them the best likelihood of being able to impute gaps with minimal computational lag time and hardware requirements for use in clinical contexts compared to tensor-based and machine learning-based methods. However, the lack of transparency in the reporting of the data and computational requirements of these algorithms make it difficult to make such conclusions with any certainty. Additionally, the lack of any comparison between method types as well as between methods makes the tradeoff between imputation accuracy, data requirements, and computational latency difficult to assess quantitatively.

### 4.1. Limitations of the Literature

An objective of this systematic review was to elucidate a leading imputation methodology for EEG missing data gap and artifacts. There were multiple methods that exhibited strong performance in the imputation of missing EEG data. However, there were several limitations in the literature that made it difficult to identify a leading method.

Broadly, the included studies demonstrated limited generalizability, calling into question the scope of applicability. Most studies used small datasets (typically fewer than 10 subjects), provided limited participant demographic information, and were only conducted on specific pathophysiological phenotypes. Factors such as age, biological sex, and disease all have the potential to impact EEG signal dynamics. Consequentially, factoring in the limited capture of physiologic dynamics in these articles across population cohorts calls into question the scope of applicability. For example, Vafidis et al. [45] reported a strong performance of the proposed gap-filling method while the patient was at rest and at the beginning of epileptiform discharge; however, this performance plummeted during epileptiform discharge due to the abrupt change in EEG signal dynamics. This highlights the necessity to broadly test imputation methodologies across a range of physiological conditions. Furthermore, because EEG exhibits strong channel-to-channel dependencies due to functional connectivity and common sources of excitation, it can be expected to see varying dynamics across behavioral states (task compared to rest). The non-stationarity and variability in the underlying electrophysiological activations further limits the generalizability of these imputation algorithms across experimental conditions. For example, there is a significant range in the performance indicated by RMSE and MAE for the algorithm(s) presented in Liu et al. (2022) [42]. Tasks being conducted while riding a stationary bike seem to result in significantly worsened imputation performance, potentially attributed to differing electrophysiological excitations or noise induced by movement. Nevertheless, the included studies depict preliminary investigations to address real-world concern in dealing with missing data.

Variations in study methodology also resulted in difficulty to accurately compare results across studies. There were significant differences in the recording methodologies that were used. Most of the included studies used scalp EEG devices for data collection; however, different commercial devices were used, with some articles not listing any details on the particular device used. There was also variance in the number of channels/electrodes used to measure EEG signals, ranging from 3 to 100, with additional differences in the sampling rates and anatomical locations of the electrodes. The spatial resolution of EEG makes it such that there can be differences in the temporal dynamics captured in recordings based on the location of the sensor. Furthermore, the proposed articles aimed to interpolate missing segments, requiring segments of signals to be artificially removed such that they could be compared to a ‘ground truth’. However, the provision of the characteristics of these gaps as well as descriptions of the gap incidence were often insufficient or missing altogether in the articles examined. The potential variability in the missing data made it difficult to accurately compare the performances of imputation methodologies between articles.

There was glaringly insufficient information reported regarding the computational requirements and latency associated with the application of these imputation methods. Only five of the sixteen articles included in this review provided any specifications for the computational hardware required to run the developed models. Furthermore, only three of the sixteen articles reported any computational time or lag time associated with the developed models. The timely reconstruction of missing data segments in EEG signals is paramount for the utility of EEG signals specifically in contexts where they are used to guide clinical decisions. As such, computational lag time associated with reconstruction as well as computational requirements are fundamental to the applications of these methods. Additionally, the computational hardware requirements to run the algorithms for imputation are critical to understand, as they guide the feasibility of implementation and, in the future, may have bearing on the design of future workstations in clinical, research, and diagnostic settings. Many of the included articles did not provide substantial information regarding training or testing datasets, making it difficult to understand how much data is required to generate accurate imputations. Additionally, there was considerable heterogeneity in terms of the characteristics of a given missing data gap. Given the importance of the aforementioned information, the following should be considered standard in reporting for EEG gap imputation methods: (1) how much data was required to perform an imputation of a particular duration, (2) what computational processor was used to complete the imputation (i.e., CPU/GPU, other hardware requirements), and (3) the real-time applicability or lag time required to perform the imputation, as these all have bearing on the utility and the ability to implement the developed gap imputation methods.

### 4.2. Limitations of Review

There were some limitations associated with the scope of this review. Exclusion criteria for the articles in this review included the requirement that the article be published in English, introducing a potential language bias. There was an additional limitation that the search was initially conducted on 24 September 2024 and updated on 16 June 2025, with studies after this date not being included in the review. Thus, this might not reflect the most recent studies published afterward. Additionally, while the search included BIOSIS, SCOPUS, EMBASE, PubMed, and Cochrane Library, intended to encapsulate all indexed articles in this domain, it is possible that articles could have been missed. The initial search was conducted to encapsulate all missing data gap imputation methods for cerebral bio-signals; however, the large volume of included articles necessitated splitting the review into two works. A priori filtering was therefore required to include only cerebral electrophysiological signal gap imputation. While detailed screening was conducted to limit any selection bias, the initial search would have yielded fewer articles if only electrophysiological missing data gap imputation methods had been the focus of the original search. The lack of a homogeneous study type, with variations in sample size and differences in metrics of result evaluation, may have resulted in inadequate or insufficient conclusions made due to a lack of information sufficient to facilitate a proper meta-analysis of the literature. Finally, while each of the articles that were included in this review have been published in peer-reviewed academic journals, a comprehensive risk of bias assessment was not formally conducted. As such, the results presented for the methods developed as well as the conclusions made could have been affected by biased datasets, potential flaws in the study methodologies, selectively reporting results, or issues with the reproducibility and generalizability of these methods.

### 4.3. Recommendations and Future Directions

Several methods outlined in this review display strong imputation performance for missing EEG segments. As was highlighted in the above sections, it remains difficult to describe a single leading methodology due to the heterogeneity of the study types as well as information missing from the source articles that accurately describes the potential for clinical applications. As such, it is recommended that a more robust study be conducted that thoroughly evaluates the performance of different imputation methods.

Future study should involve data collection from sizeable and physiologically diverse populations. The signal recording methodologies followed should be chosen to maximize the generalizability of the imputation algorithms developed. There should be adequate variance in the number of channels/electrodes used and sampling rates. Further, details of the device(s) used for recording as well as the anatomical locations of electrodes should be provided. Missing data characteristics should be well-documented, including the number of segments and their duration. Further, where possible, participants should be assigned a series of instructions, including performing movement tasks, motor imagery tasks, and resting. The various stages of the recordings should be well-reported to differentiate tasks and rest. Computational latency and the potential for the real-time utility of future imputation algorithms should be evaluated, documenting computational requirements to achieve them.

Given the breadth of applications of EEG monitoring, it is difficult to describe a single overarching framework for the prioritization of elements of future methods. Certain aspects such as real-time utility and computational simplicity are critical for the live-time use of data in clinical electrophysiological and depth of sedation monitoring. In such instances, live-time information is imperative, and computational resources may be limited. As a result, the ideal method developed for such circumstances may prioritize simplicity over accuracy in EEG signal imputation. In specific research or diagnostic contexts, real-time utility and computational complexity may be less of an issue, whereas precise imputation is paramount. In such contexts, the accuracy of imputation is more vital. These different requirements for distinctly different applications of EEG signal imputation are why it is vital to conduct more exhaustive and transparent studies on EEG imputation methodologies such that considerations based on applications can be more easily evaluated in the likely context that a single leading method is not generalizable across all applications. Transparency in the algorithm requirements are also potentially vital in specifications for the design of workstations in various contexts where EEG gap imputation may be used.

Gap imputation in EEG signals plays various important roles across clinical practice, research, and diagnostics. The findings of this review highlight that a single method is particularly difficult to elucidate due to the blurring of the various advantages and disadvantages of each method attributed to inadequate inter-comparisons of method types and transparency in data and computational requirements. Such information will be critical to clarify prior to the adoption of EEG gap imputation methods. The computational requirements and computational lag time will play vital roles in deciding where a particular method is feasible to implement. The accuracy of such methods as well as the notion of potential points of imputation inaccuracy will be paramount to understand when interpreting this information is critical to guiding clinical, diagnostic, and research decisions and conclusions.

## 5. Conclusions

This systematic scoping review was conducted across five databases to investigate the current imputation methods for missing data and artifacts in EEG signals, categorizing methods into tensor-based algorithms, machine learning and deep learning methods, and model-based and classical imputation methods. There were several methodologies in each of these categories that presented strong and effective results. However, a single leading method could not be identified, as there were limited comparisons of different methods within articles and limitations of the literature made it difficult to compare performance across articles. These limitations included many of these studies demonstrating limited generalizability due to small sample size, limited physiological variability, and differences in data recording methodologies. Furthermore, these articles poorly described missing data characteristics and computational requirements. As a result, further study is required to elucidate a leading EEG gap imputation method capable of being applied in clinical settings.

## Figures and Tables

**Table 1:** Summary of tensor-based imputation methods.

Article	Study Population	Signal Type, Electrodes and SR	Missing Data Characteristics	Description of Algorithm and Effectiveness
Solé-Casals et al. [32]	Healthy (*n* = 5) Age: N/A	EEG, 6 electrodes, 256 Hz	Missing tensor entries of 1% to 20% (entries being a point missing across a channel x time-point x trial)	This article compared four tensor-completion-based algorithms initially designed to reconstruct corrupted or missing multi-channel EEG data: CP-WOPT, 3DPB-TC, BCPF, and HaLRTC. The HaLRTC algorithm had the best performance by NRMSE, with missing entry percentages of 1%, 5%, 10%, 15%, and 20%. It achieved an NRMSE value of ~10^−2^ even at 20% missing data. Train/test split: Trained and tested on same dataset (no external validation). Computational time: N/A. Computational hardware: N/A.
Akmal et al. (2021) [34]	1. Healthy (*n* = 9) Age: N/A 2. Healthy (*n* = 1) Age: N/A	1. EEG, 3 electrodes, 250 Hz 2. EEG, 3 electrodes, 128 Hz	Structured missing data of 10% to 50%	Compared three imputation methods designed to fill absent EEG data: CPD, NMF, CP-WOPT. CP-WOPT had the best performance, achieving the lowest RME, ranging from 0.044 to 0.101 across 10–50% missing data ratios across all patients. Train/test split: Trained and tested on same dataset (no external validation). Computational time: For 10% to 50% missing data, 4.5 to 5.1 s (CPD), 6.1 to 6.7 s (CP-WOPT), and 3.1 to 4 s (NMF). Computational hardware: Windows 8, i3 processor, 6 GB of RAM.
Akmal and Zubair [33]	1. Healthy (*n* = 1) Age: 25 2. Healthy (*n* = 9) Age: 24.7 ± 3.3 years	1. EEG, 3 electrodes, 128 Hz 2. EEG, 3 electrodes, 250 Hz	Structured missing data of 10% to 50%	Compared three imputation methods designed to fill absent EEG data: NMF, CPD, CP-WOPT. The CP-WOPT algorithm had the best performance across 10–50% missing data ratios, achieving an RME of 0.04 to 0.1 on dataset 1 and 0.04 to 0.12 on dataset 2 across missing data ratios of 10% to 50%. Train/test split: Trained and tested on same dataset (no external validation). Computational time: N/A. Computational hardware: Windows 8, i3 processor, 6 GB of RAM.
Duan et al. [35]	Healthy (*n* = 15) Age: 22–40 years	EEG, 11 electrodes, 256 Hz	Simulated missing data of variable duration (0.125 to 2 s) and number of channels affected (5 to 100)	Compared four tensor completion methods designed to fill absent EEG data: BCPF, HaLRTC, CP-WOPT, and STDC. The STDC method outperformed the other tensor completion methods and the baseline method, consistently achieving LNRMSE > 2.0 across both across 5 to 100 time series affected by missing segments of 0.25 and 0.5 s. STDC also outperformed other methods when number of time series affected was kept constant at 10 and 20, when missing signal segment duration ranged from 0.125 s to 2.00 s. Train/test split: Trained and tested on same dataset (no external validation). Computational time: N/A. Computational hardware: N/A.
Akmal et al. (2023a) [36]	1. Healthy (*n* = 9) Age: N/A 2. Healthy (*n* = 1) Age: N/A	1. EEG, 3 electrodes, 250 Hz 2. EEG, 3 electrodes, 128 Hz	Structured missing data of 10% to 50%	Compared six imputation methods designed to fill absent EEG data: X-WCP, BGCP, NTF, CPD, TD, CP-WOPT. The tensor-based X-WCP had the best performance compared to the other tensor completion methods, achieving RME of 0.15 at missing data ratios of 50% at the lowest computational run time. Train/test split: Trained and tested on same dataset (no external validation). Computational time: 3.36 s, what this time was studying was not specified but it indicates faster performance compared to other methods. Computational hardware: N/A.
Akmal (2023b) [37]	1. Healthy (*n* = 9) Age: N/A 2. Healthy (*n* = 1) Age: N/A 3. Healthy (*n* = 4) Age: N/A	1. EEG, 3 electrodes, 250 Hz 2. EEG, 3 electrodes, 128 Hz 3. EEG, 4 electrodes, 500 Hz	Structured missing data of 10% to 50%	Compared three imputation methods designed to fill absent EEG data: NMF, CPD, CP-WOPT. The tensor-based CP-WOPT had the best performance, achieving RME of around 0.14 at missing data ratios of 50%. Train/test split: Trained and tested on same dataset (no external validation). Computational time: N/A. Computational hardware: NVIDIA Quadro k620 GPU with 256 GB of RAM.
Cui et al. [38]	Unspecified participant status (*n* = 5) Age: N/A	EEG, 8 electrodes, 256 Hz	Data missing ratio of 10% to 70%	This article presented the BCPF tensor-based method, which achieved a low RME of <0.1 when the data missing ratio was under 0.7. This method was designed to fill in missing EEG data. Train/test split: Trained and tested on same dataset (no external validation). Computational time: N/A. Computational hardware: N/A.

3DPB-TC = 3D Patch-based Tensor Completion, BCPF = Bayesian Canonical Polyadic Factorization, CP = Canonical Polyadic, CPD = CP Decomposition, CP-WOPT = CP Decomposition Factorization with Weighted Optimization Method, EEG = Electroencephalogram, HaLRTC = High-Accuracy Low-Rank Tensor Completion, Hz = Hertz, LNRMSE = Logarithm Normalized Root Mean Square Error, N/A = Not Available, NMF = Nonnegative Matrix Factorization, NRMSE = Normalized Root Mean Square Error, NTF = Non-negative Tensor Factorization, RME = Relative Mean Error, SR = Sampling Rate, STDC = Simultaneous Tensor Decomposition and Completion, TD = Turker Decomposition, X-WCP = Xavier-initialized Weighted Canonical Polyadic Decomposition.

**Table 2:** Summary of machine learning and deep learning imputation methods.

Article	Population Information	Signal Type, Electrodes and SR	Missing Data Characteristics	Description of Algorithm and Effectiveness
Liu et al. (2024) [39]	Epilepsy patients (*n* = 10) Age: N/A	EEG, N/A, N/A	Missing segments are randomly simulated in each dataset at fixed missing rates 10%, 30%, and 50%	The presented algorithm was a BiLSTMELM-ENSAIT model designed to impute missing EEG values, which was compared to an Im-BiLSTM model. The BiLSTM-ELM had the best performance, achieving an RMSE of 0.234 and R^2^ of 0.723 compared to the ‘ground-truth’ signal at missing rate of 50%. Train/test split: 8/2. Computational time: N/A. Computational hardware: Windows 10, 13th Gen Intel(R) Core(TM) i9-13900KF 3.00 GHz CPU, 64 GB of RAM, NVIDIA GeForce RTX 4090 GPU.
Leng et al. [40]	N/A (*n* = N/A) Age: N/A	Scalp EEG, 14 electrodes, N/A	Missing data is introduced completely at random with rates from 20% to 80% (step size of 10%)	This article presented the SCAPC-GAIN algorithm, which was designed to impute missing EEG data, comparing it to GAIN, PC-GAIN, and MA-GAIN algorithms. The SCAPC-GAIN had the best performance, achieving an RMSE of 0.3407 at a missing data rate of 50%. Train/test split: 5-fold cross-validation. Computational time: N/A. Computational hardware: N/A.
Ren and Pan [41]	N/A (*n* = 9) Age: N/A	EEG, 22 electrodes, 250 Hz	Missing data introduced with durations of 500, 1000, and 2000 milliseconds	The presented algorithm was the TFCMI-CNN-LSTM model, designed to impute missing EEG data that was removed as a result of noise contamination. The best performance of the algorithm was when correlated channels were used as features, achieving mean RMSE, MAE, and SpR values of 3.512, 2.787, and 0.828, respectively, across the nine electrodes for which imputation was applied, with missing data durations of 500 milliseconds. Train/test split: N/A. Computational time: N/A. Computational hardware: N/A.
Liu et al. (2022) [42]	1. N/A, (*n* = 12), Age: N/A (split into 1a and 1b based on experimental conditions) 2. N/A, (*n* = 26), Age: N/A 3. N/A, (*n* = 9), Age: N/A	(1) EEG, N/A, 256 Hz down sampled to 128 Hz (2) EEG, N/A, N/A down sampled to 128 Hz (3) EEG, 22 electrodes, 250 Hz down sampled to 128 Hz	Synthesized missing data gaps at incidence of 10%	The presented model was the SRI-EEG model, designed to impute missing EEG data. This model achieved the best performance across all datasets, achieving RMSE values of 88.192 ± 1.70 for dataset 1a, 12.142 ± 0.45 for dataset 1b, 69.985 ± 0.85 for dataset 2, and 0.889 ± 0.01 for dataset 3 on 10% missing data incidence, outperforming the use of the trial mean value, KNN, ICA, MRNN, and BRITS algorithms. Train/test split: Datasets (1) and (2) indicated to have been split 80–20%; no specifications for dataset (3). There was 15% of the training set indicated to have been left out for validation. Computational time: N/A. Computational hardware: N/A.

BiLSTM = Bi-directional Long Short-Term Memory Network Long Short-Term Memory, BiLSTMELM-ENSAIT = Bi-directional Long Short-Term Memory Network Extreme Learning Machine-Elastic Net Self-Attention Inception Time Network, BRITS = Bidirectional Recurrent Imputation for Time Series, EEG = Electroencephalogram, GAIN = Generative Adversarial Imputation Networks, Hz = Hertz, ICA = Independent Component Analysis, Im-LSTM = Imputation Long Short-Term Memory, KNN = k-Nearest Neighbor, MA-GAIN = Missingness Augmentation Generative Adversarial Imputation Networks, MAE = Mean Absolute Error, MRNN = Multi-Directional Recurrent Neural Networks, N/A = Not Available, PC-GAIN = Pseudo-label Conditional Generative Adversarial Imputation Networks, R^2^ = Coefficient of Determination, SCAPC-GAIN = Spectral Clustering Augmentation Pseudo-label Conditional Generation Adversarial Imputation Networks, SR = Sampling Rate, SpR = Spearman Rank, SRI-EEG = State-Based Recurrent Imputation for EEG, TFCMI-CNN-LSTM = Time–Frequency Cross-Mutual Information Convolutional Neural Network Long Short-Term Memory.

**Table 3:** Summary of model-based and classical methods.

Article	Population Information	Signal Type, Electrodes and SR	Missing Data Characteristics	Description of Algorithm and Effectiveness
Ayyoubi et al. [43]	Refractory epilepsy (*n* = 3) Age: 12, 19, and 33 years	iEEG, 32 electrodes, (1000 Hz)	Simulated 10% packet loss at random points in data	This article compared three classical imputation algorithms used to impute missing segments simulating ‘packet loss’: PVR, SI, and LI. LI and SI had far superior performance to PVR. They indicated accurate recovery of low-frequency components of the data in all three patients, but performance worsened with higher-frequency components as defined by PSD values. However, LI and PI showed PSD values close to that of the raw data, with an approximate maximum difference of 2 to 3 dB even at 500 Hz. Train/test split: N/A. Computational time: Not provided but indicated to be low. Computational hardware: N/A.
Kanemura et al. [44]	Healthy (*n* = 9) Age: 5–16 years	EEG, 22 electrodes (250 Hz)	Channels randomly selected to have data replaced with NaN values	This article presented the statistical modeling-based MAR method for imputation and compared it to NNI and LI for imputing missing values. The MAR demonstrated the lowest mean of RMSE among these three methods. which was 2.143 across subjects. Train/test split: N/A. Computational time: N/A. Computational hardware: N/A.
Vafidis et al. (2019) [45]	Absence epilepsy (*n* = 1) Age: 37 years	TMS-Scalp EEG (TMS-EEG), 60 electrodes (1450 Hz)	Induced TMS artifacts; however, methods included from this article treated corrupted signal as NaN values	This article presented the ‘gap-filling’ data-driven model-based imputation method, initially conceived to impute segments corrupted by TMS artifacts. However, this method functions by replacing the corrupted segment with a gap before imputation. It compared the performance of this method to the shape-preserving piecewise cubic spline interpolation method, as well as FastICA and PCA-based methods; however, only the gap-filling and shape-preserving piecewise cubic spline interpolation methods were considered. The other methods relied on an underlying EEG signal for reconstruction. The gap-filling had competitive performance during resting conditions and at the beginning of epileptiform discharge, achieving RMSE values of 13.95 and 20.23 for each dataset compared to the shape-preserving piecewise cubic spline interpolation, which achieved RMSE values of 13.72 and 20.54. However, the shape-preserving piecewise cubic spline interpolation had similar performance for those datasets and had superior performance during the epileptiform discharge, achieving an RMSE value of 18.23 compared to 44.29 for the gap-filling method. Train/test split: N/A. Computational time: N/A. Computational hardware: N/A.
Xiong et al. [46]	N/A (*n* = N/A) Age: N/A Only one of three datasets was included due to an absence of a ‘ground truth’ in the other two	TMS-EEG, 64 electrodes, (N/A)	Induced TMS artifacts which were treated as NaN by included methods	This article presented the model-based 3DARQHI algorithm, which is a signal recombination algorithm relying upon rational Hermite interpolation. This method was applied to segments that had been removed where TMS pulse artifacts had been corrupting the segment. It was compared to PCHI, PCA, FastICA, gap-filling, and SOUND algorithms; however, PCA and FastICA were not considered due to their reliance on underlying EEG signal. The 3DARQHI had the best performance in terms of the lowest RMSE in the EEG dataset for which a true ‘ground truth’ existed, improved from 2498.42 to 0.34. Train/test split: N/A. Computational time: Ran 3-periods (1200 timepoints) of 64 channel EEG data in 1.45 s. Computational hardware: HP Z840 workstation, 16-core Intel Xeon E5-2620 CPU, 32 GB of memory.
Kim et al. [47]	1. Spasmodic epilepsy (*n* = 3) Age: N/A 2. Unspecified participants status (*n* = 6) Age: N/A 3. Unspecified participants status (*n* = 3) Age: N/A	(1) EEG, 100 electrodes, (N/A) (2) EEG, 31 electrodes, (N/A) (3) EEG, 64 electrodes (N/A)	Missing data are artificially introduced across all datasets at random rates (5%, 10%, 15% for accuracy analyses) to simulate missingness for testing	This article presents the model-based iEMPCA algorithm for filling gaps in EEG data. The performance of this algorithm was compared two algorithms: robust EMPCA and MSVD. The iEMPCA outperformed the other two methods in datasets 1 and 2, achieving RMSE values of 0.0344 and 0.0163, respectively. However, it underperformed the other two methods using dataset 3, where Robust EMPCA and MSVD had RMSE values of 0.0262 and 0.0300, respectively, compared to the 0.0313 obtained by the iEMPCA algorithm. Train/test split: N/A. Computational time: N/A. Computational hardware: N/A.

3DARQHI = 3D Adaptive Rational Quadratic Hermite Interpolation, EEG = Electroencephalogram, EMPCA = Expectation Maximization Principal Component Analysis, Hz = Hertz, ICA = Fast Independent Component Analysis, iEEG = Intracranial Electroencephalogram, iEMPCA = Incremental EMPCA, LI = Linear Interpolation, MAR = Multivariate Autoregressive, MSVD = Missing Value Singular Value Decomposition, N/A = Not Available, NaN = Not A Number, NNI = Nearest-Neighbor Interpolation, PCA = Principal Component Analysis, PCHI = Piecewise Cubic Hermite Interpolation, PSD = Power Spectral Density, PVR = Previous Value Replacement, RMSE = Root Mean Square Error, SI = Spline Interpolation, SOUND = Structured Output Unsupervised Denoising, SR = Sampling Rate, TMS = Transcranial Magnetic Stimulation.

## Data Availability

All relevant data are contained in the manuscript and the extraction tables provided in the Supplementary Materials (Tables S2–S4) or are publicly available at their respective data source.

## Supplementary Materials

The following supporting information can be downloaded at: https://www.mdpi.com/article/10.3390/s26082431/s1, Table S1: PRISMA ScR Checklist; Table S2: Tensor Based Methods; Table S3: Machine Learning and Deep Learning Methods; Table S4: Model-Based and Classical Methods.

## References

[1] Britton J.W., Frey L.C., Hopp J.L., Korb P., Koubeissi M.Z., Lievens W.E., Pestana-Knight E.M., St Louis E.K. (2016). Electroencephalography (EEG): An Introductory Text and Atlas of Normal and Abnormal Findings in Adults, Children, and Infants.

[2] Rayi A., Murr N.I. (2025). Electroencephalogram. StatPearls [Internet].

[3] Lisgaras C.P., De La Prida L.M., Bertram E., Cunningham M., Henshall D., Liu A.A., Gnatkovsky V., Balestrini S., De Curtis M., Galanopoulou A.S. (2025). The Role of Electroencephalography in Epilepsy Research—From Seizures to Interictal Activity and Comorbidities. Epilepsia.

[4] Huang Z., Yang Y., Ma Y., Dong Q., Su J., Shi H., Zhang S., Hu L. (2025). EEG Detection and Recognition Model for Epilepsy Based on Dual Attention Mechanism. Sci. Rep..

[5] Tatum W.O., Rubboli G., Kaplan P.W., Mirsatari S.M., Radhakrishnan K., Gloss D., Caboclo L.O., Drislane F.W., Koutroumanidis M., Schomer D.L. (2018). Clinical Utility of EEG in Diagnosing and Monitoring Epilepsy in Adults. Clin. Neurophysiol..

[6] Chaddad A., Wu Y., Kateb R., Bouridane A. (2023). Electroencephalography Signal Processing: A Comprehensive Review and Analysis of Methods and Techniques. Sensors.

[7] Bitar R., Khan U.M., Rosenthal E.S. (2024). Utility and Rationale for Continuous EEG Monitoring: A Primer for the General Intensivist. Crit. Care.

[8] Rosenthal E.S. (2021). Seizures, Status Epilepticus, and Continuous EEG in the Intensive Care Unit. Continuum.

[9] Westover M.B., Shafi M.M., Bianchi M.T., Moura L.M.V.R., O’Rourke D., Rosenthal E.S., Chu C.J., Donovan S., Hoch D.B., Kilbride R.D. (2015). The Probability of Seizures during EEG Monitoring in Critically Ill Adults. Clin. Neurophysiol..

[10] Ch’ang J., Claassen J. (2017). Seizures in the Critically Ill. Handbook of Clinical Neurology.

[11] Pandian J.D., Cascino G.D., So E.L., Manno E., Fulgham J.R. (2004). Digital Video-Electroencephalographic Monitoring in the Neurological-Neurosurgical Intensive Care Unit: Clinical Features and Outcome. Arch. Neurol..

[12] Rubinos C., Alkhachroum A., Der-Nigoghossian C., Claassen J. (2020). Electroencephalogram Monitoring in Critical Care. Semin. Neurol..

[13] Zhang K., Zang S., Li Z., Wang Z., Ji Y., Zhao H. (2025). EEG-Based Detection for Depth of Sedation Using Spectro-Temporal Information in ICU Patients. Proceedings of the 2025 28th International Conference on Computer Supported Cooperative Work in Design (CSCWD), Compiegne, France, 5–7 May 2025.

[14] Rasulo F.A., Hopkins P., Lobo F.A., Pandin P., Matta B., Carozzi C., Romagnoli S., Absalom A., Badenes R., Bleck T. (2023). Processed Electroencephalogram-Based Monitoring to Guide Sedation in Critically Ill Adult Patients: Recommendations from an International Expert Panel-Based Consensus. Neurocrit. Care.

[15] Siuly S., Li Y., Zhang Y. (2016). EEG Signal Analysis and Classification.

[16] Dabbabi T., Bouafif L., Cherif A. (2023). A Review of Non Invasive Methods of Brain Activity Measurements via EEG Signals Analysis. Proceedings of the 2023 IEEE International Conference on Advanced Systems and Emergent Technologies (IC_ASET), Hammamet, Tunisia, 29 April–1 May 2023.

[17] Shah A., Mittal S. (2014). Invasive Electroencephalography Monitoring: Indications and Presurgical Planning. Ann. Indian Acad. Neurol..

[18] Jiang X., Bian G.-B., Tian Z. (2019). Removal of Artifacts from EEG Signals: A Review. Sensors.

[19] Tamburro G., Fiedler P., Stone D., Haueisen J., Comani S. (2018). A New ICA-Based Fingerprint Method for the Automatic Removal of Physiological Artifacts from EEG Recordings. PeerJ.

[20] Labate D., La Foresta F., Mammone N., Morabito F.C., Bassis S., Esposito A., Morabito F.C. (2015). Effects of Artifacts Rejection on EEG Complexity in Alzheimer’s Disease. Advances in Neural Networks: Computational and Theoretical Issues.

[21] Stone D.B., Tamburro G., Fiedler P., Haueisen J., Comani S. (2018). Automatic Removal of Physiological Artifacts in EEG: The Optimized Fingerprint Method for Sports Science Applications. Front. Hum. Neurosci..

[22] Mathias S.V., Bensalem-Owen M. (2019). Artifacts That Can Be Misinterpreted as Interictal Discharges. J. Clin. Neurophysiol..

[23] Yildirim S. (2025). An Overview of ECG Artifact Detection in EEG Signals. J. Cardiovasc. Med. Cardiol..

[24] Kaya İ, Asadpour V. (2022). A Brief Summary of EEG Artifact Handling. Artificial Intelligence.

[25] Radüntz T., Scouten J., Hochmuth O., Meffert B. (2017). Automated EEG Artifact Elimination by Applying Machine Learning Algorithms to ICA-Based Features. J. Neural Eng..

[26] Urigüen J.A., Garcia-Zapirain B. (2015). EEG Artifact Removal—State-of-the-Art and Guidelines. J. Neural Eng..

[27] Islam M.K., Rastegarnia A., Yang Z. (2016). Methods for Artifact Detection and Removal from Scalp EEG: A Review. Neurophysiol. Clin./Clin. Neurophysiol..

[28] Schalk G., McFarland D.J., Hinterberger T., Birbaumer N., Wolpaw J.R. (2004). BCI2000: A General-Purpose Brain-Computer Interface (BCI) System. IEEE Trans. Biomed. Eng..

[29] Goldberger A.L., Amaral L.A.N., Glass L., Hausdorff J.M., Ivanov P.C., Mark R.G., Mietus J.E., Moody G.B., Peng C.-K., Stanley H.E. (2000). PhysioBank, PhysioToolkit, and PhysioNet: Components of a New Research Resource for Complex Physiologic Signals. Circulation.

[30] Page M.J., McKenzie J.E., Bossuyt P.M., Boutron I., Hoffmann T.C., Mulrow C.D., Shamseer L., Tetzlaff J.M., Akl E.A., Brennan S.E. (2021). The PRISMA 2020 Statement: An Updated Guideline for Reporting Systematic Reviews. BMJ.

[31] PRISMA Extension for Scoping Reviews (PRISMA-ScR): Checklist and Explanation. https://www.acpjournals.org/doi/epdf/10.7326/M18-0850.

[32] Solé-Casals J., Caiafa C.F., Zhao Q., Cichocki A. (2018). Brain-Computer Interface with Corrupted EEG Data: A Tensor Completion Approach. Cogn. Comput..

[33] Akmal M., Zubair S. (2022). Artificial Neural Network-Based Framework for Improved Classification of Tensor-Recovered EEG Data. IEEE Sens. J..

[34] Akmal M., Zubair S., Alquhayz H. (2021). Classification Analysis of Tensor-Based Recovered Missing EEG Data. IEEE Access.

[35] Duan F., Jia H., Zhang Z., Feng F., Tan Y., Dai Y., Cichocki A., Yang Z., Caiafa C.F., Sun Z. (2021). On the Robustness of EEG Tensor Completion Methods. Sci. China Technol. Sci..

[36] Akmal M., Abid M.I., Bakr M.A., Khan M.O., Saeed N. (2023). A Fast Convergent and Robust Classifier for Multi-Way Corrupted Eeg Signals. Multimed. Tools Appl..

[37] Akmal M. (2023). Tensor Factorization and Attention-Based CNN-LSTM Deep-Learning Architecture for Improved Classification of Missing Physiological Sensors Data. IEEE Sens. J..

[38] Cui G., Gui L., Zhao Q., Cichocki A., Cao J. (2016). Bayesian CP Factorization of Incomplete Tensor for EEG Signal Application. Proceedings of the 2016 IEEE International Conference on Fuzzy Systems (FUZZ-IEEE), Vancouver, BC, Canada, 24–29 July 2016.

[39] Liu J.-J., Yao J.-P., Liu J.-H., Wang Z.-Y., Huang L. (2024). Missing Data Imputation and Classification of Small Sample Missing Time Series Data Based on Gradient Penalized Adversarial Multi-Task Learning. Appl. Intell..

[40] Leng Q., Liu W., Meng X. (2023). Spectral Clustering Augmentation Pseudo-Label Conditional Generation Adversarial Imputation Networks for Multivariate Time-Series Missing Data. Proceedings of the 2023 9th International Conference on Systems and Informatics (ICSAI), Changsha, China, 16–18 December 2023.

[41] Ren B., Pan Y. (2023). Extracting and Supplementing Method for EEG Signal in Manufacturing Workshop Based on Deep Learning of Time–Frequency Correlation. J. Intell. Manuf..

[42] Liu Y., Höllerer T., Sra M. (2022). SRI-EEG: State-Based Recurrent Imputation for EEG Artifact Correction. Front. Comput. Neurosci..

[43] Ayyoubi A.H., Fazli Besheli B., Quach M.M., Gavvala J.R., Goldman A.M., Swamy C.P., Bartoli E., Curry D.J., Sheth S.A., Francis D.J. (2024). Benchmarking Signal Quality and Spatiotemporal Distribution of Interictal Spikes in Prolonged Human iEEG Recordings Using CorTec Wireless Brain Interchange. Sci. Rep..

[44] Kanemura A., Cheng Y., Kaneko T., Nozawa K., Fukunaga S. (2018). Imputing Missing Values in EEG with Multivariate Autoregressive Models. Proceedings of the 2018 40th Annual International Conference of the IEEE Engineering in Medicine and Biology Society (EMBC), Honolulu, HI, USA, 18–21 July 2018.

[45] Vafidis P., Kimiskidis V.K., Kugiumtzis D. (2019). Evaluation of Algorithms for Correction of Transcranial Magnetic Stimulation-Induced Artifacts in Electroencephalograms. Med. Biol. Eng. Comput..

[46] Xiong H., Di Y., Liu J., Han Y., Zheng Y. (2023). A Three-Dimensional Adaptive Rational Interpolation Algorithm for Removing TMS-EEG Pulse Artifacts. Physiol. Meas..

[47] Kim S.H., Yang H.J., Ng K.S. (2011). Incremental Expectation Maximization Principal Component Analysis for Missing Value Imputation for Coevolving EEG Data. J. Zhejiang Univ.-Sci. C.

[48] Lagerlund T.D., Sharbrough F.W., Jack C.R., Erickson B.J., Strelow D.C., Cicora K.M., Busacker N.E. (1993). Determination of 10–20 System Electrode Locations Using Magnetic Resonance Image Scanning with Markers. Electroencephalogr. Clin. Neurophysiol..

[49] Bullock T., Cecotti H., Giesbrecht B. (2015). Multiple Stages of Information Processing Are Modulated during Acute Bouts of Exercise. Neuroscience.

[50] Margaux P., Emmanuel M., Sébastien D., Olivier B., Jérémie M. (2012). Objective and Subjective Evaluation of Online Error Correction during P300-Based Spelling. Adv. Hum.-Comput. Interact..

[51] Tangermann M., Müller K.-R., Aertsen A., Birbaumer N., Braun C., Brunner C., Leeb R., Mehring C., Miller K.J., Müller-Putz G.R. (2012). Review of the BCI Competition IV. Front. Neurosci..

[52] Goodfellow I.J., Pouget-Abadie J., Mirza M., Xu B., Warde-Farley D., Ozair S., Courville A., Bengio Y. (2014). Generative Adversarial Networks. arXiv.

[53] Epileptology. https://www.ukbonn.de/en/epileptology/.

[54] Free EEG Data Database Freely ERP Data Publicly. https://sccn.ucsd.edu/~arno/fam2data/data_description_for_nitrc.html.

[55] BCI Competition II. https://www.bbci.de/competition/ii/.

[56] Yun B.I. (2021). C 2 Weighted Piecewise Rational Interpolation. Appl. Math. Comput..

[57] Zhao L., Chai T., Cong Q. (2006). Operating Condition Recognition of Pre-Denitrification Bioprocess Using Robust EMPCA and FCM. Proceedings of the 2006 6th World Congress on Intelligent Control and Automation.

[58] Troyanskaya O., Cantor M., Sherlock G., Brown P., Hastie T., Tibshirani R., Botstein D., Altman R.B. (2001). Missing Value Estimation Methods for DNA Microarrays. Bioinformatics.

[59] Chuang C.-H., Chang K.-Y., Huang C.-S., Jung T.-P. (2022). IC-U-Net: A U-Net-Based Denoising Autoencoder Using Mixtures of Independent Components for Automatic EEG Artifact Removal. NeuroImage.

[60] Leach S., Sousouri G., Huber R. (2023). ‘High-Density-SleepCleaner’: An Open-Source, Semi-Automatic Artifact Removal Routine Tailored to High-Density Sleep EEG. J. Neurosci. Methods.

[61] Gransier R., Guérit F., Carlyon R.P., Wouters J. (2021). Frequency Following Responses and Rate Change Complexes in Cochlear Implant Users. Hear. Res..

[62] De Cheveigné A., Arzounian D. (2018). Robust Detrending, Rereferencing, Outlier Detection, and Inpainting for Multichannel Data. NeuroImage.

[63] Suárez-Revelo J.X., Ochoa-Gómez J.F., Tobón-Quintero C.A., Figueroa-García J.C., Villegas J.G., Orozco-Arroyave J.R., Maya Duque P.A. (2018). Validation of EEG Pre-Processing Pipeline by Test-Retest Reliability. Applied Computer Sciences in Engineering.

[64] Cox R., Weber F.D., Van Someren E.J.W. (2024). Customizable Automated Cleaning of Multichannel Sleep EEG in SleepTrip. Front. Neuroinform..

[65] Korhonen R.J., Hernandez-Pavon J.C., Metsomaa J., Mäki H., Ilmoniemi R.J., Sarvas J. (2011). Removal of Large Muscle Artifacts from Transcranial Magnetic Stimulation-Evoked EEG by Independent Component Analysis. Med. Biol. Eng. Comput..

[66] Leske S., Dalal S.S. (2019). Reducing Power Line Noise in EEG and MEG Data via Spectrum Interpolation. NeuroImage.

[67] Mutanen T.P., Metsomaa J., Liljander S., Ilmoniemi R.J. (2018). Automatic and Robust Noise Suppression in EEG and MEG: The SOUND Algorithm. NeuroImage.

[68] Bahmer A., Pieper S., Baumann U. (2018). Evaluation of an Artifact Reduction Strategy for Electrically Evoked Auditory Steady-State Responses: Simulations and Measurements. J. Neurosci. Methods.

[69] Khatun S., Mahajan R., Morshed B.I. (2016). Comparative Study of Wavelet-Based Unsupervised Ocular Artifact Removal Techniques for Single-Channel EEG Data. IEEE J. Transl. Eng. Health Med..

[70] Waddell C., Pratt J.A., Porr B., Ewing S. (2009). Deep Brain Stimulation Artifact Removal Through Under-Sampling and Cubic-Spline Interpolation. Proceedings of the 2009 2nd International Congress on Image and Signal Processing, Tianjin, China, 17–19 October 2009.

[71] De Cheveigné A. (2016). Sparse Time Artifact Removal. J. Neurosci. Methods.

[72] Lamer A., Jeanne M., Marcilly R., Kipnis E., Schiro J., Logier R., Tavernier B. (2016). Methodology to Automatically Detect Abnormal Values of Vital Parameters in Anesthesia Time-Series: Proposal for an Adaptable Algorithm. Comput. Methods Programs Biomed..

[73] Liew S.-H., Choo Y.-H., Low Y.F., Hung J.C., Yen N.Y., Hui L. (2019). Data Imputation in EEG Signals for Brainprint Identification. Frontier Computing.

[74] Touil M., Bahatti L., Elmagri A., Lekova A. (2020). EEG Signal Cleaning for Drowsiness Detection. Proceedings of the 2020 International Conference on Electrical and Information Technologies (ICEIT), Rabat, Morocco, 4–7 March 2020.

[75] Pulferer H.S., Müller-Putz G.R. (2022). Continuous Error Processing during a Closed-Loop 2D Tracking Task. Curr. Dir. Biomed. Eng..

[76] Badnjević A., Gurbeta Pokvić L. (2024). MEDICON’23 and CMBEBIH’23: Proceedings of the Mediterranean Conference on Medical and Biological Engineering and Computing (MEDICON) and International Conference on Medical and Biological Engineering (CMBEBIH), September 14–16, 2023, Sarajevo, Bosnia and Herzegovina—Volume 1: Imaging, Engineering and Artificial Intelligence in Healthcare.

[77] Deng X., Zhu J., Yang S. (2021). SFE-Net: EEG-Based Emotion Recognition with Symmetrical Spatial Feature Extraction. Proceedings of the 29th ACM International Conference on Multimedia, Virtual Event, 20–24 October 2021.

[78] Olkkonen H., Pesola P., Olkkonen J., Valjakka A., Tuomisto L. (2002). EEG Noise Cancellation by a Subspace Method Based on Wavelet Decomposition. Med. Sci. Monit..

[79] Ciarleglio A., Petkova E., Harel O. (2022). Elucidating Age and Sex-Dependent Association Between Frontal EEG Asymmetry and Depression: An Application of Multiple Imputation in Functional Regression. J. Am. Stat. Assoc..

[80] Jas M., Engemann D., Raimondo F., Bekhti Y., Gramfort A. (2016). Automated Rejection and Repair of Bad Trials in MEG/EEG. Proceedings of the 2016 International Workshop on Pattern Recognition in Neuroimaging (PRNI), Trento, Italy, 22–24 June 2016.

[81] Jas M., Engemann D.A., Bekhti Y., Raimondo F., Gramfort A. (2017). Autoreject: Automated Artifact Rejection for MEG and EEG Data. NeuroImage.

[82] Larson E., Taulu S. (2018). Reducing Sensor Noise in MEG and EEG Recordings Using Oversampled Temporal Projection. IEEE Trans. Biomed. Eng..

[83] Somervail R., Cataldi J., Stephan A.M., Siclari F., Iannetti G.D. (2023). Dusk2Dawn: An EEGLAB Plugin for Automatic Cleaning of Whole-Night Sleep Electroencephalogram Using Artifact Subspace Reconstruction. Sleep.

[84] Xie J., Li C., Li N., Li P., Wang X., Gao D., Yao D., Xu P., Yin G., Li F. (2021). Robust Autoregression with Exogenous Input Model for System Identification and Predicting. Electronics.

[85] Saba-Sadiya S., Alhanai T., Liu T., Ghassemi M.M. (2020). EEG Channel Interpolation Using Deep Encoder-Decoder Networks. Proceedings of the 2020 IEEE International Conference on Bioinformatics and Biomedicine (BIBM), Seoul, Republic of Korea, 16–19 December 2020.

[86] Kobler R.J., Sburlea A.I., Mondini V., Muller-Putz G.R. (2019). HEAR to Remove Pops and Drifts: The High-Variance Electrode Artifact Removal (HEAR) Algorithm. Proceedings of the 2019 41st Annual International Conference of the IEEE Engineering in Medicine and Biology Society (EMBC), Berlin, Germany, 23–27 July 2019.

[87] Mahmud S., Hossain M.S., Chowdhury M.E.H., Reaz M.B.I. (2023). MLMRS-Net: Electroencephalography (EEG) Motion Artifacts Removal Using a Multi-Layer Multi-Resolution Spatially Pooled 1D Signal Reconstruction Network. Neural Comput. Appl..

[88] Naik S., Dehaene-Lambertz G., Battaglia D. (2023). Repairing Artifacts in Neural Activity Recordings Using Low-Rank Matrix Estimation. Sensors.

[89] Narmada A., Shukla M.K. (2023). A Novel Adaptive Artifacts Wavelet Denoising for EEG Artifacts Removal Using Deep Learning with Meta-Heuristic Approach. Multimed. Tools Appl..

[90] Roy V., Shukla S. (2017). Effective EEG Motion Artifacts Elimination Based on Comparative Interpolation Analysis. Wirel. Pers. Commun..

[91] Castellanos N.P., Makarov V.A. (2006). Recovering EEG Brain Signals: Artifact Suppression with Wavelet Enhanced Independent Component Analysis. J. Neurosci. Methods.

[92] Dora C., Biswal P.K. (2020). Correlation-Based ECG Artifact Correction from Single Channel EEG Using Modified Variational Mode Decomposition. Comput. Methods Programs Biomed..

[93] Krishnaveni V., Jayaraman S., Aravind S., Hariharasudhan V., Ramadoss K. (2006). Automatic Identification and Removal of Ocular Artifacts from EEG Using Wavelet Transform. Meas. Sci. Rev..

[94] Singh B., Wagatsuma H. (2019). Two-Stage Wavelet Shrinkage and EEG-EOG Signal Contamination Model to Realize Quantitative Validations for the Artifact Removal from Multiresource Biosignals. Biomed. Signal Process. Control.

[95] Sweeney K.T., McLoone S.F., Ward T.E. (2013). The Use of Ensemble Empirical Mode Decomposition with Canonical Correlation Analysis as a Novel Artifact Removal Technique. IEEE Trans. Biomed. Eng..

[96] Chen X., Chen Q., Zhang Y., Wang Z.J. (2019). A Novel EEMD-CCA Approach to Removing Muscle Artifacts for Pervasive EEG. IEEE Sens. J..

[97] Zhang Z., Yu X., Rong X., Iwata M. (2022). A Novel Multimodule Neural Network for EEG Denoising. IEEE Access.

[98] Bahadur I.N., Boppana L. (2024). Efficient Architecture for Ocular Artifacts Removal from EEG: A Novel Approach Based on DWT-LMM. Microelectron. J..

[99] Chen S., Wang B. (2024). An Attention-UNet Model to Remove Artifacts from Single-Channel EEG. Proceedings of the 2024 17th International Congress on Image and Signal Processing, BioMedical Engineering and Informatics (CISP-BMEI), Shanghai, China, 26–28 October 2024.

[100] Chuang C.-H., Chang K.-Y., Huang C.-S., Bessas A.-M. (2025). Augmenting Brain-Computer Interfaces with ART: An Artifact Removal Transformer for Reconstructing Multichannel EEG Signals. NeuroImage.

[101] Mahmud S., Chowdhury M.E.H., Kiranyaz S., Al Emadi N., Tahir A.M., Hossain M.S., Khandakar A., Al-Maadeed S. (2024). Restoration of Motion-Corrupted EEG Signals Using Attention-Guided Operational CycleGAN. Eng. Appl. Artif. Intell..

[102] Corley I.A., Huang Y. (2018). Deep EEG Super-Resolution: Upsampling EEG Spatial Resolution with Generative Adversarial Networks. Proceedings of the 2018 IEEE EMBS International Conference on Biomedical & Health Informatics (BHI), Las Vegas, NV, USA, 4–7 March 2018.

[103] Svantesson M., Olausson H., Eklund A., Thordstein M. (2021). Virtual EEG-Electrodes: Convolutional Neural Networks as a Method for Upsampling or Restoring Channels. J. Neurosci. Methods.

[104] Liu R., Wang Z., Qiu J., Wang X. (2023). Assigning Channel Weights Using an Attention Mechanism: An EEG Interpolation Algorithm. Front. Neurosci..

[105] Dong L., Zhao L., Zhang Y., Yu X., Li F., Li J., Lai Y., Liu T., Yao D. (2021). Reference Electrode Standardization Interpolation Technique (RESIT): A Novel Interpolation Method for Scalp EEG. Brain Topogr..

[106] Luo T., Fan Y., Chen L., Guo G., Zhou C. (2020). EEG Signal Reconstruction Using a Generative Adversarial Network with Wasserstein Distance and Temporal-Spatial-Frequency Loss. Front. Neuroinform..

[107] Tveitstøl T., Tveter M., Pérez T. A.S., Hatlestad-Hall C., Yazidi A., Hammer H.L., Hebold Haraldsen I.R.J. (2024). Introducing Region Based Pooling for Handling a Varied Number of EEG Channels for Deep Learning Models. Front. Neuroinform..

[108] Khan M.A. (2024). A Comparative Study on Imputation Techniques: Introducing a Transformer Model for Robust and Efficient Handling of Missing EEG Amplitude Data. Bioengineering.

[109] Zhang J., Yin P. (2019). Multivariate Time Series Missing Data Imputation Using Recurrent Denoising Autoencoder. Proceedings of the 2019 IEEE International Conference on Bioinformatics and Biomedicine (BIBM), San Diego, CA, USA, 18–21 November 2019.

