# Supplementary material for: Missing Data Gap Imputation Methods in Electroencephalogram (EEG) Signals: A Systematic Scoping Review
Source: Sensors (Basel). 2026 Apr 15;26(8):2431. doi: 10.3390/s26082431 (PMC13119862; doi:10.3390/s26082431)
Supplement: Supplementary file 1 [file sensors-26-02431-s001.zip › sensors-4237632-supplementary.pdf]

6 Supplementary File S1

Table S1. PRISMA ScR Checklist

Section and Topic	Item #	Checklist item	Location where item is reported
**Title**			
Title	1	Identify the report as a systematic review.	Pg. 1 (Title)
**Abstract**			
Abstract	2	See the PRISMA 2020 for Abstracts checklist.	Pg. 1-2 (Abstract)
**Introduction**			
Rationale	3	Describe the rationale for the review in the context of existing knowledge.	Pg. 2-3 (Section 1)
Objectives	4	Provide an explicit statement of the objective(s) or question(s) the review addresses.	Pg. 3 (Section 1)
**Methods**			
Eligibility criteria	5	Specify the inclusion and exclusion criteria for the review and how studies were grouped for the syntheses.	Pg. 4 (Section 2.2)
Information sources	6	Specify all databases, registers, websites, organisations, reference lists and other sources searched or consulted to identify studies. Specify the date when each source was last searched or consulted.	Pg. 4-5 (Section 2.3)
Search strategy	7	Present the full search strategies for all databases, registers and websites, including any filters and limits used.	Pg. 4-5 (Section 2.3) + Pg. 35-36 (Supplementary File S2)
Selection process	8	Specify the methods used to decide whether a study met the inclusion criteria of the review, including how many reviewers screened each record and each report retrieved, whether they worked independently, and if applicable, details of automation tools used in the process.	Pg. 5 (Section 2.4)
Data collection process	9	Specify the methods used to collect data from reports, including how many reviewers collected data from each report, whether they worked independently, any processes for obtaining or confirming data from study investigators, and if applicable, details of automation tools used in the process.	Pg. 5-6 (Section 2.5)
Data items	10a	List and define all outcomes for which data were sought. Specify whether all results that were compatible with each outcome domain in each study were sought (e.g. for all measures, time points, analyses), and if not, the methods used to decide which results to collect.	Pg. 5-6 (Section 2.5)
	10b	List and define all other variables for which data were sought (e.g. participant and intervention characteristics, funding sources). Describe any assumptions made about any missing or unclear information.	N/A
Study risk of bias assessment	11	Specify the methods used to assess risk of bias in the included studies, including details of the tool(s) used, how many reviewers assessed each study and whether they worked independently, and if applicable, details of automation tools used in the process.	All articles published in academic journals, as such, biases were assumed to have been screened; Pg. 6 (Section 2.6)
Effect	12	Specify for each outcome the effect measure(s) (e.g. risk ratio, mean	N/A

measures		difference) used in the synthesis or presentation of results.	
Synthesis methods	13a	Describe the processes used to decide which studies were eligible for each synthesis (e.g. tabulating the study intervention characteristics and comparing against the planned groups for each synthesis (item #5)).	N/A
	13b	Describe any methods required to prepare the data for presentation or synthesis, such as handling of missing summary statistics, or data conversions.	N/A
	13c	Describe any methods used to tabulate or visually display results of individual studies and syntheses.	All data items for each method were tabulated and are included in Supplementary File S3 (Table C.1-C.3)
	13d	Describe any methods used to synthesize results and provide a rationale for the choice(s). If meta-analysis was performed, describe the model(s), method(s) to identify the presence and extent of statistical heterogeneity, and software package(s) used.	N/A
	13e	Describe any methods used to explore possible causes of heterogeneity among study results (e.g. subgroup analysis, meta-regression).	N/A
	13f	Describe any sensitivity analyses conducted to assess robustness of the synthesized results.	N/A
Reporting bias assessment	14	Describe any methods used to assess risk of bias due to missing results in a synthesis (arising from reporting biases).	Pg. 29 (Section 4.2)
Certainty assessment	15	Describe any methods used to assess certainty (or confidence) in the body of evidence for an outcome.	N/A
**Results**			
Study selection	16a	Describe the results of the search and selection process, from the number of records identified in the search to the number of studies included in the review, ideally using a flow diagram.	Pg. 6-7 (Section 3)
	16b	Cite studies that might appear to meet the inclusion criteria, but which were excluded, and explain why they were excluded.	Pg. 21-22 (Section 4)
Study characteristics	17	Cite each included study and present its characteristics.	Pg. 7-21 (Section 3.1 - 3.3)
Risk of bias in studies	18	Present assessments of risk of bias for each included study.	All articles published in academic journals, as such, biases were assumed to have been screened
Results of individual studies	19	For all outcomes, present, for each study: (a) summary statistics for each group (where appropriate) and (b) an effect estimate and its precision (e.g. confidence/credible interval), ideally using structured tables or plots.	Supplementary File S3 (Table S2-S4)
Results of syntheses	20a	For each synthesis, briefly summarise the characteristics and risk of bias among contributing studies.	All articles published in academic journals, as such, biases were assumed to have been screened
	20b	Present results of all statistical syntheses conducted. If meta-analysis was done, present for each the summary estimate and its precision (e.g. confidence/credible interval) and measures of statistical heterogeneity. If	No statistical synthesis conducted

		comparing groups, describe the direction of the effect.	
	20c	Present results of all investigations of possible causes of heterogeneity among study results.	Pg. 7-21 (Section 3.1 - 3.3)
	20d	Present results of all sensitivity analyses conducted to assess the robustness of the synthesized results.	Pg. 7-21 (Section 3.1 - 3.3)
Reporting biases	21	Present assessments of risk of bias due to missing results (arising from reporting biases) for each synthesis assessed.	Pg. 24 (Section 4.2)
Certainty of evidence	22	Present assessments of certainty (or confidence) in the body of evidence for each outcome assessed.	N/A
**DISCUSSION**			
Discussion	23a	Provide a general interpretation of the results in the context of other evidence.	Pg. 21-23 (Section 4)
	23b	Discuss any limitations of the evidence included in the review.	Pg. 23-24 (Section 4.1)
	23c	Discuss any limitations of the review processes used.	Pg. 24 (Section 4.2)
	23d	Discuss implications of the results for practice, policy, and future research.	Pg. 24-25 (Section 4.3)
**OTHER INFORMATION**			
Registration and protocol	24a	Provide registration information for the review, including register name and registration number, or state that the review was not registered.	Review was not registered
	24b	Indicate where the review protocol can be accessed, or state that a protocol was not prepared.	Protocol not prepared
	24c	Describe and explain any amendments to information provided at registration or in the protocol.	N/A
Support	25	Describe sources of financial or non-financial support for the review, and the role of the funders or sponsors in the review.	Pg. 25 (Funding)
Competing interests	26	Declare any competing interests of review authors.	Pg. 26 (Conflicts of Interest)
Availability of data, code and other materials	27	Report which of the following are publicly available and where they can be found: template data collection forms; data extracted from included studies; data used for all analyses; analytic code; any other materials used in the review.	N/A

7 Supplementary File S2

The following search string was used to search five databases (BIOSIS, SCOPUS, EMBASE, PubMed, and Cochrane Library) examining keywords, abstracts, and titles indexed in these databases:

("AMP" OR "ARI" OR "arterio-jugular oxygen content " OR "Arterio–venous difference" OR "arteriovenous difference in oxygen" OR "Autonomics" OR "Autoregulation index" OR "Autoregulatory reserve" OR "AVDO2" OR **"BIS"** OR **"Bispectral "** OR "Blood Flow Velocities" OR "Blood Flow Velocity" OR "Bowman perfusion" OR "Brain Autoregulation " OR "Brain Blood Flow " OR "Brain metabolism" OR "Brain metabolites" OR "Brain Perfusion" OR "brain tissue oxygen tension" OR "Brain tissue oxygenation" OR "CA" OR "CBF" OR "CBFV" OR "CBFx" OR "CBV" OR "Cerebral artery flow velocity" OR "Cerebral Autoregulation" OR "Cerebral Blood Flow" OR "Cerebral Blood Flow Index " OR "Cerebral blood flow velocity" OR "Cerebral blood volume" OR "Cerebral circulation" OR "Cerebral Flow metabolism" OR "Cerebral hemodynamics" OR "Cerebral homeostasis" OR "Cerebral metabolic rate for oxygen " OR "Cerebral metabolism" OR "Cerebral microcirculation" OR "Cerebral microdialysis" OR "Cerebral Oximetry index " OR "Cerebral Oximetry Index " OR "Cerebral perfusion" OR "Cerebral Perfusion Pressure" OR "Cerebral pressure" OR "Cerebral pressure autoregulation" OR "Cerebral spinal reserve capacity" OR "Cerebral vascular reactivity" OR "Cerebral vascular resistance" OR "Cerebral vasculature" OR "Cerebral vasoconstriction" OR "Cerebral vasodilation" OR "Cerebral vasomotor responsiveness" OR "Cerebral Vasoreactivity" OR "Cerebral Vessel Diameter" OR "Cerebrovascular autoregulation" OR "Cerebrovascular control" OR "Cerebrovascular Function" OR "Cerebrovascular reactivity" OR "CMD" OR "CMRO2" OR "Compensatory reserve " OR "Cortical blood flow" OR "Cortical laser doppler" OR "Cortical perfusion " OR "COx" OR "COx-a" OR "CPP" OR "Dax" OR "DCS" OR **"dEEG"** OR "**dense array electroencephalography"** OR "Deoxyhemoglobin Index " OR "Diastolic flow index" OR "Diastolic Flow Index " OR "Diffuse Correlation Spectroscopy" OR "Diffusion Weighted Imaging index" OR "DWI" OR "Dx" OR "Dx-a" OR "Dynamic autoregulatory index" OR **"EcOG"** OR **"EEG"** OR **"Electrocortical activity"** OR **"Electrocorticography"** OR **"Electroencephalography"** OR **"Electrophysiological monitoring"** OR **"Electrophysiology"** OR "End-tidal CO2 " OR "EtCO2" OR "Flow velocity " OR "FV" OR "HbOx " OR "HbOx-a" OR "HBx" OR "Hbx-a" OR "Heart rate" OR "Hemedex" OR "hemoglobin volume index" OR "Hemoglobin Volume Index " OR "HVx" OR "ICP" OR "Induced Pressure Reactivity Index " OR "Intracranial pressure" OR "iPRx" OR "Jugular bulb saturation " OR "Jugular venous oxygen saturation" OR "Kety–Schmidt technique" OR "L-PRx" OR "Laser Doppler flowmetry " OR "Laser-Doppler Index " OR "LAx" OR "LDF" OR "LDx" OR "Licox" OR "Long Pressure Reactivity Index " OR "Low-Frequency Autoregulation Index " OR "Lx" OR "Lx-a" OR "Mean flow index" OR "Mean Flow Index " OR "Mean transit time " OR "Median arterial pressure" OR "MTT" OR "Multi-scale entropy" OR "Mx" OR "Mx-a" OR "Near infrared spectroscopy " OR "Near-infrared spectrometry" OR "Neurovascular autoregulation" OR "Neurovascular coupling" OR "Neurovascular reactivity" OR "Neurovent-PTO" OR "NIRS" OR "OEF" OR "OHT" OR "orthostatic hypotension test " OR "ORx" OR "oxygen extraction fraction " OR "Oxygen Reactivity Index " OR "Oxyhemoglobin Index " OR "Parenchymal brain tissue oxygen" OR "Parenchymal thermal diffusion" OR "PAx" OR "PAx" OR "PbtO2" OR "Perfusion-weighted imaging index" OR "Peripheral oxygen saturation " OR

"PPR" OR "Pressure Reactivity index" OR "Projection pursuit regression" OR "PRx" OR "PRx55-15" OR "Pulsatile Reactivity index " OR "Pulsatility Index" OR "Pulse Amplitude Index " OR "Pulse amplitude of ICP" OR "RAC" OR "RAP" OR "regional cerebral oxygen saturation" OR "Respiratory dynamics" OR "rSO2" OR **"sEEG"** OR "Signal entropy" OR "SjO2" OR "Spatially resolved NIRS" OR "SpO2" OR **"stereo electroencephalography"** OR "SvjO2" OR "Sx" OR "Sx-a" OR "Systolic flow index" OR "Systolic Flow Index " OR "TCCS" OR "TCD" OR "TCDT" OR "TCDx" OR "TDx" OR "TF" OR "TFA" OR "tHbx" OR "Thermal Diffusion " OR "Thermal diffusion catheter" OR "Thermal diffusion probe" OR "Thigh cuff deflation technique " OR "THRT" OR "THx" OR "THx-a" OR "Time to peak" OR "tissue hemoglobin index" OR "Tissue Oxygen Index " OR "Tissue Oxygenation index" OR "Total Hemoglobin Index " OR "Tox" OR "TOx " OR "TOx-a" OR "Transcranial color-coded duplex sonography " OR "Transcranial Doppler" OR "Transcranial doppler index " OR "Transcranial Doppler Sonography" OR "Transfer function analysis" OR "transient hyperemic response test" OR "TTP" OR "Vascular reactivity" OR "Wavelet Cerebral Oximetry Index " OR "Wavelet Hemoglobin Volume Index " OR "Wavelet Pressure Reactivity Index " OR "wCOx" OR "wHVx" OR "wPRx")

NOTE: **Bolded** terms correspond to cerebral electrophysiological signals that were considered for inclusion in this review

AND

("Curve estimation methods" OR "Data deficiency" OR "Data fitting techniques" OR "Data gaps" OR "Data omission" OR "Data reconstruction methods" OR "Data voids" OR "Estimation algorithms" OR "Function approximation strategies" OR "Incomplete data" OR "Interpolation methods" OR "Interpolation techniques" OR "Lost data" OR "Missing data" OR "Noise suppression" OR "Null data" OR "Sampling techniques" OR "Signal reconstruction" OR "Smoothing approaches" OR "Unavailable data" OR "Value approximation techniques" OR "Value prediction techniques

8 Supplementary File S3

Table S2. Tensor Based Methods

Reference	Subject information	Signal type and duration	Missing data characteristics	Location and number of sensors	Imputation technique used	Methods compared	Effectiveness	Results of study	Limitations
Solé-Casals et al. [32]	Five healthy subjects (age: N/A)<br><br>Eight sessions on different days are recorded. Task duration is approximately 6 seconds; procedure is repeated 80 to 170 times<br><br>Conducting motor imagery tasks<br><br>Data available: Laboratory for Advanced Brain Signal Processing, BSI-RIKEN, Japan in collaboration with Shanghai Jio Tong University, China [110].	EEG, sampling rate is 256 Hz<br><br>Recorded by g.tec (g.USBamp)).<br><br>Band-pass filtered between 2 and 30 Hz with a notch filter at 50 Hz.	EEG data sets are reinforced with intentional corrupted/missing entries for two situations: random missing entries and random missing channels.<br><br>1%, 5%, 10%, 15%, and 20% of missing samples on the whole tensor are considered.	6 electrodes included C3, Cz, C4, CP3, CPz, and CP4.	Four tensor completion-based techniques were evaluated to reconstruct corrupted multi-channel EEG signal segments:<br>1. CP-WOPT uses the CP algorithms [111] for factorization , models the known data points using weighted least squares regression, optimizes factor matrices simultaneously using a first-order optimization, and fills in missing data points after fitting the global model for tensor data [112].<br>2. 3DPB-TC divides a 3D tensor into overlapping sub-tensors, vector representations of sub-tensors are assumed to follow sparse representations (i.e., low-rank). Sparse Tucker decomposition is applied to similar patches to construct small local dictionaries matrices to reconstruct missing segments [113].<br>3. BCPF uses CP factorization, automatically determining the global tensor rank using hierarchical sparsity inducing priors of latent factor matrices following a Bayesian inference framework, this is used to reconstruct missing segments [114].<br>4. HaLRTC uses the generalized trace norm of a tensor based on the convex combination of trace norms corresponding to the different unfolded mode networks, optimized using the ADMM algorithm [115] and uses the resultant tensor to fill in missing entries [116].	Comparison between presented methods	NRMSE was calculated between estimated and actual EEG values.<br><br>Across missing-data ratios of 1%, 5%, 10%, 15%, and 20%, all tensor-completion algorithms (CP-WOPT, 3DPB-TC, BCPF, HaLRT) had strong imputation performance by NRMSE. The results had to be extracted from a plot; however, it was clear that all four tensor methods had NRMSE values below 10^{-1} across all missing data ratios.<br><br>HaLRTC had the lowest NRMSE values of the tensor-completion methods for missing entries. It achieved a NRMSE value of $\sim 10^{-2}$ even at 20% missing data.	HaLRTC had the best performance of the tensor-based methods; however, all showed improvement in imputation of missing EEG data points.<br><br>However, CP-WOPT performed better in full channel imputation (not the focus of this study)	Only examines missing entries of entire tensor, not necessarily representative of missing time-series segments.<br><br>Low subject size and channel number used.<br><br>CP-WOPT and 3DPB-TC require parameter tuning.<br><br>Methods are tested offline, with no mention of real-time utility or computational cost.<br><br>EEG is assumed to be stationary.<br><br>Requires sufficient data to examine global latent patterns, a significant limitation for real-time utility.<br><br>Not validated on independent dataset

					CP-WOPT and 3DPB-TC algorithms had rank optimized using range of parameter values to minimize NRMSE based on dataset with 10% of channels removed.<br><br>No mention of computer specifications used.				Only applied on entire dataset, no mention of real-time imputation.
Akmal et al. (2021) [34]	Two datasets are used:<br>(1) One female (age: 25 years)<br><br>Performing imagery hand motions<br><br>Experiment run 7 times with 40 trials each<br><br>Data available: Dataset III from BCI competition II) [117]<br><br>(2) Nine healthy participants with normal vision (age: 24.7±3.3 years)<br><br>Performing imagery hand motions<br><br>5 sessions each<br><br>Data available: Dataset 2b from BCI competition IV [118].<br><br>Combined training and testing datasets are1152 x 3 x 140 (samples x channels x trials)	For (1): EEG, sampling rate is 128 Hz.<br><br>For (2): EEG, sampling rate is 250 Hz.	Missing data (10%-50%) is introduced only in test data by removing continuous segments from Cz, C3+C4, or all three channels (C3, Cz, C4)	For (1) three Ag/AgCl electrodes places over C3, Cz, and C4.<br><br>For (2) three bipolar recordings with C3, Cz, C4.	This method evaluates two EEG missing data imputation methods:<br>1. CPD iteratively optimizes an objective function that measures the difference between actual and the reconstructed tensors to determine factor matrices that best describe the data.<br>2. CP-WOPT iteratively optimizes a weighted objective function that ignores missing points when conducting tensor optimization.<br><br>The reconstructed data was used to try to conduct classification tasks, but this was not relevant to this review.<br><br>Computational hardware included: Windows 8 operating system, i3 processor, and 6 GB RAM.	Compared to NMF [119].	In CP-WOPT across both datasets, mean RME increases slightly from 0.044 (10% missing) to 0.101 (50% missing), indicating the best results compared to CPD and NMF methods. These methods had RME increase from 0.5 to above 0.6.<br><br>Three-way analysis of variance with Tukey's honest significant difference confirms CP-WOPT's significantly better recovery performance ($P<0.05$).<br><br>Time for reconstruction to recover 10% to 50% was: 4.5 to 5.1 seconds for CPD, 6.1 to 6.7 seconds for CP-WOPT, and 3.1 to 4 seconds for NMF for 1152 x 3 x 140 datapoints.	CP-WOPT is the most effective imputation method by RME.<br><br>Also, the missing percentage has a positive relationship with execution time.<br><br>NMF had the fastest execution time among compared methods.	Study is limited to EEG signals from three electrodes (C3, Cz, C4).<br><br>Tensor rank not determined automatically.<br><br>Somewhat idealized missing data, entire slices removed systematically from data.<br><br>Not validated on independent dataset.<br><br>Only applied on entire dataset, no mention of real-time imputation.

Akmal and Zubair [33]	Two datasets are used:<br>(1) Nine healthy, right-handed participants with normal vision<br><br>No mention of task/rest<br><br>No mention of session duration<br><br>Data available: BCI Competition IV Dataset 2b [118].<br><br>(2) EEG data from is acquired from 1 normal participant<br><br>No mention of task/rest<br><br>No mention of session duration<br><br>Data available: BCI Competition II Dataset III [117].<br><br>Combined training and testing datasets are1152 x 3 x 140 (samples x channels x trials)	For (1): EEG, sampling rate is 250 Hz<br><br>For (2): EEG, sampling rate is 128 Hz.	Missing data (10-50%) is introduced in a structured manner using slices of data at time point.	For both datasets, the electrodes used are C3, Cz, and C4.	The proposed tensor-based methods include CPD and CP-WOPT which are described in this Table with previous entry. Authors present the results from the same dataset presented by Akmal et al. [34].<br><br>The reconstructed data was used to try to conduct classification tasks, but this was not relevant to this review.<br><br>Dataset: 1152 x 3 x 140 (samples x channels x trials)<br><br>Computational hardware included: Windows 8 operating system, i3 processor, and 6 GB RAM.	Compares tensor-based imputation methods NMF [119] and CPD	RME was calculated between the observed and reconstructed tenor data.<br><br>On the BCI competition IV dataset, RME slightly increases from 0.04 to 0.1 as missing data increases from 10% to 50%. CP-WOPT algorithm was able to recover 90% of the missing data at 50% missing.<br><br>On the BCI competition II dataset, RME increases slightly from 0.04 to 0.12 as the missing data rate increases from 10% to 50%.	The ANN with CP-WOPT framework effectively improves classification accuracy in the presence of structured missing EEG data.	Data only records from C3, Cz, and C4 positions.<br><br>Evaluation was performed on dataset with 9 and 1 participants.<br><br>Method was not compared to other algorithms.<br><br>Not validated on independent dataset.<br><br>Idealized missing data, no missing segments.<br><br>Only applied on entire dataset, no mention of real-time imputation.
Duan et al. (2021) [35]	15 participants<br><br>Participants were asked to perform a series of six movements and one resting state	EEG downsampled to 256 Hz.	Missing data entries in the input tensor are created by randomly selecting location and duration of missing entries They vary the number of time	11 channels are used (F3, Fz, F4, FCz, C1, Cz, C2, CPz, P3, Pz, P4) according to the international 10-20 system.	This article compared four different tensor completion algorithms:<br>1. CP-WOPT method developed by Acar et al. [112], previously described.<br>2. BCPF method developed by Zhao et al. [114], previously described.<br>3. STDC developed by Chen et al. [121] which also relies on rank	These methods are compared to basic one-dimensional linear interpolation, considered baseline for	STDC has the highest reconstruction accuracy. It evaluates using the LNRMSE (-$\log_{10}$ of NRMSE) between original and reconstructed EEG tensors.	STDC is most stable across all simulations and performs better than baseline interpolation and other TCMs	Only used single metric (LNRMSE), as opposed to more usually used RMSE and NMRSE.<br><br>STDC likely requires entire

	50 of 60 trials selected, each approximately 3 seconds<br><br>Data available: Ofner et al. [120]		series with missing entries (N_{mCT}) from 5 to 100 (step size of 5) and the time length of the missing data (N_{mTL}) from 0.125 to 2.00 seconds (step size of 0.125 seconds), referred to as Simulation I in the article.<br><br>Keeping N_{mTL} constant at 0.25 seconds and 0.5 seconds, respectively and varying N_{mCT} referred to in this manuscript as Simulation I(a).<br><br>Keeping N_{mCT} constant at 10 and 20, respectively and varying N_{mTL} referred to in this manuscript as Simulation I(b).		minimization; however, this method uses Tucker Decomposition. This method decomposes the optimized tensor into four components, found using the maximum a posteriori strategy relying on factor priors. The resultant optimized tensor is used to estimate missing data.<br>4. HaLRTC method developed by Liu et al. (2013) [116], previously described.<br><br>No mention of computer specifications used.	LNRMSE calculations	Results for Simulation I(a) indicate that the STDC method outperformed the other tensor completion methods and the baseline method, consistently achieving LNRMSE > 2.0 across both constant N_{mTL}.<br><br>Results for Simulation I(b) indicate that the STDC method outperformed the other methods, achieving LRMSE between ~2.1 to ~1.8 when duration of missing segments varied from 0.125 seconds to 2.00 seconds affecting 10 or 20 time series.	under varying missing data.<br><br>Relatively consistent performance across all methods despite changing number of time-series affected.	datasets for global tensor construction, similar to CP-WOPT and HaLRTC, potentially limiting real-time applicability.<br><br>Not validated on independent dataset.<br><br>Only applied on entire dataset, no mention of real-time imputation.
Akmal et al. (2023a) [36]	Two datasets are used:<br>(1) Nine right-handed healthy adults with normal vision<br><br>No mention of task/rest<br><br>No mention of session duration	For (1) EEG sampling rate at 250 Hz (bandpass 0.5-100 Hz, 50 Hz notch).<br><br>For (2) EEG sampling rate at 128 Hz (bandpass 0.5-30 Hz).	Structured missing data simulates in chunks at 10% - 50% levels with step size 10%.	3 EEG channels (C3, Cz, C4) are used for both datasets	The proposed method is the X-WCP algorithm, which is a modified version of the CP-WOPT algorithm, aiming to improve its speed of convergence to an optimized tensor. As opposed to using random values for weights, Xavier initialization is used, setting weights near global minima (variance=1/p). Factor matrices are optimized via gradient descent (max 1000 iterations, 10000 evaluations).	The method is compared to CPD, CP-WOPT, TD, BGCP, and NTF.	For the RME between recovered and original EEG:<br>X-WCP = 0.15<br>CP-WOPT = 0.18<br>BGCP = 0.23<br>NTF = 0.26<br>CP = 0.36<br>TD = 0.37.<br><br>X-WCP has the lowest RME at all missing data	X-WCP outperforms all compared methods in accuracy of data recovery and computational efficiency and substantially improves classification performance on	Only 3 EEG channels are used.<br><br>Two datasets with small participant numbers.<br><br>TD, BGCP, and NTF not introduced in detail.

	Data available: BCI competition IV dataset 2b [118].<br><br>(2) One healthy adult<br><br>No mention of task/rest<br><br>No mention of session duration<br><br>Data available: BCI competition II dataset III [117].<br><br>Combined training and testing datasets are 1152 x 3 x 140 (samples x channels x trials)				Dataset: 1152 x 3 x 140 (samples x channels x trials)<br><br>No mention of computer specifications used.<br><br>The reconstructed data was used to try to conduct classification tasks, but this was not relevant to this review.		levels (RME=0.15 at 50% missing), outperforming CP (0.36), CP-WOPT (0.18), TD (0.37), BGCP (0.23), and NTF (0.26).<br><br>X-WCP is 38.7%-51.6% faster in runtime compared to other methods, mean runtime of 3.36 seconds.	recovered data compared to partial data.	Not validated on independent dataset.<br><br>Only applied on entire dataset, no mention of real-time imputation.
Akmal (2023b) [37]	Three datasets are used, but one is EMG data so not included: (1) Nine healthy, right-handed participants with normal vision<br><br>No mention of task/rest<br><br>No mention of session duration<br><br>Data available: BCI competition IV dataset 2b [118].<br><br>(2) One normal participant	For (1): EEG, sampling rate of 250 Hz (bandpass filter 0.5-100 Hz, notch filter 50 Hz).<br><br>For (2): EEG, sampling rate of 128 Hz (bandpass filter 0.5-30 Hz).	Structured missing data introduced resulting in 10% to 50% missing	For (1) and (2) 3 electrodes are used (C3, Cz, C4).	The proposed method is the CP-WOPT method developed by Acar et al. [112], previously described.<br><br>Computational hardware included: NVIDIA Quadro k620 GPU with 256 GB of RAM	The method is compared to NMF and CPD	CP-WOPT has lowest relative mean error (RME) across all datasets:<br>For dataset (1):<br>At missing value 10%, NMF = 0.2, CP = 0.27, CP-WOPT = 0.05.<br>At missing value 20%, NMF = 0.32, CP = 0.33, CP-WOPT = 0.06.<br>At missing value 30%, NMF = 0.35, CP = 0.39, CP-WOPT = 0.08.<br>At missing value 40%, NMF = 0.42, CP = 0.43, CP-WOPT = 0.09.<br>At missing value 50%, NMF = 0.48, CP = 0.48, CP-WOPT = 0.12.<br><br>For dataset (2):	For the overall performance, CP-WOPT shows best among NMF and CP in recovering missing data.	Simulated missingness might not represent real world signal loss.<br><br>Not validated on independent dataset.<br><br>Only applied on entire dataset, no mention of real-time imputation.<br><br>Small number of participants in each group.

	No mention of task/rest<br><br>No mention of session duration<br><br>Data available: BCI competition II dataset III [117].						At missing value 10%, NMF = 0.1, CP = 0.57, CP-WOPT = 0.04.<br>At missing value 20%, NMF = 0.24, CP = 0.61, CP-WOPT = 0.07.<br>At missing value 30%, NMF = 0.33, CP = 0.68, CP-WOPT = 0.01.<br>At missing value 40%, NMF = 0.57, CP = 0.72, CP-WOPT = 0.13.<br>At missing value 50%, NMF = 0.61, CP = 0.76, CP-WOPT = 0.16.<br><br>As the missing value increases, CP-WOPT is scalable, and its performance does not degrade as abruptly.		
Cui et al. [38]	Real EEG data from 5 participants bases<br><br>ERP P300 experiment.<br>No mention of session duration<br><br>Data available: N/A	EEG, sampling rate of 256 Hz	Missing data is simulated uniformly and randomly using a binary mask with ratios set at 10% to 70% (in 10% increments), reflecting real world EEG signal loss from poor electrode contact.	Eight electrodes are used positioning at Fz, Cz, Cp5, Pz, Cp6, Po7, Oz and Po8. Reference electrodes are A1 and A2.	The proposed method is Bayesian CP factorization of incomplete tensors based on the methodology outlined by Zhao et al. [114]. Rather than treating rank as a fixed parameter, BCPF applies sparsity inducing priors to factor matrices. These priors are then tuned through Bayesian inference for automated tensor rank selection.<br><br>Dataset: 180 x 8 x 800 (samples x channels x trials)<br><br>No mention of computer specifications used.	No numerical comparison of other methods is provided.	Relative standard error remains <0.1 for missing data ratios up to 70%.<br><br>The method performs poorly when the missing ratio >70%, indicating a threshold beyond which recovery is unreliable.<br><br>The classification accuracy of recovered EEG signals is approximately 60% when the missing data ratio is <50%.	The method is shown to be effective for EEG data imputation under moderate to high levels of missingness.	Performance declines with very high missing data ratios (>70%).<br><br>Small patient cohort.

3D = Three Dimensional, 3DPB-TC = 3D Patch-based Tensor Completion, ADMM = Alternating Direction Method of Multiplier, ANN = Artificial Neural Network, ANN = Artificial Neural Network, BCPF = Bayesian CP Factorization, BCPF = Bayesian CP Factorization, BGCP = Bayesian Gaussian CP, CCP = Canonical Correlation Patterns, CP = Canonical Polyadic, CP = Canonical Polyadic, CP-WOPT = Canonical Polyadic Decomposition Factorization with Weighted Optimization, CP-WOPT = Canonical Polyadic Weighted Optimization, CPD = Canonical Polyadic Decomposition, CPD = Canonical Polyadic Decomposition, EEG = Electroencephalogram, ERP = Event Related Potentials, HaLR = High Accuracy Low-rank Tensor Completion, HaLRTC = High-Accuracy Low-Rank Tensor Completion, LNRMSE = Logarithm Normalized Root Mean Square Error, MAP = Maximum a Posteriori, MI = Motor Imagery, NCG = Non-linear Conjugate Gradient, N/A = Not Available, NmCT = Number of Time Series With Missing Entries, NmTL = Time Length of The Gap, NMF = Nonnegative Matrix Factorization, NRMSE = Normalized Root Mean

Square Error, NTF = Non-negative Tensor Factorization, RME = Relative Mean Error, RME = Relative Mean Error, STDC = Simultaneous Tensor Decomposition and Completion, TD = Tucker Decomposition, TRCA = Task-Related Component Analysis, X-WCP = Xavier-initialized Weighted Canonical Polyadic Decomposition

Table S3. Machine Learning and Deep Learning Methods

Reference	Subject information	Signal type	Missing data characteristics	Location and number of sensors	Imputation technique used	Methods compared	Effectiveness	Results of study	Limitations
Liu et al. (2024) [39]	Four datasets are used; however, only one was comprised of EEG data<br><br>(2) 10 epilepsy patients<br><br>No mention of task/rest<br><br>No mention of session duration, total of 123,000 datapoints used<br><br>Data available: EEG Epilepsy Datasets from Neurology and Sleep Centre, New Delhi [122]	For (2) EEG, unspecified sampling rate.	Missing segments are randomly simulated in each dataset at fixed missing rates of omission at 10%, 30%, and 50%.	Number and location of electrodes are not specified for any dataset.	The proposed method is a BiLSTMELM-ENSAIT model for imputing missing time series values. The BiLSTM captures both past and future temporal dependencies as well as long-term dependencies. The ELM layer added to the BiLSTM model accelerates training and reduces model overfitting by randomly initializing input weights and only training output weights. Imputation using the BiLSTMELM model is triggered by a missing time-series value.<br><br>Training: testing split was 8:2<br><br>The imputation module is integrated into a multitask model for simultaneous imputation and classification; however, this was not the focus of this review.<br><br>Computational hardware included: Windows 10, the computer CPU was 13th Gen Intel(R) Core(TM) i9-13900KF 3.00 GHz, RAM was 64.00 GB, GPU was NVIDIA GeForce RTX 4090	The method is compared to Im-BiLSTM [123] and AJ-RNN [124] models; however, the AJ-RNN was only used for classification, not imputation.	For dataset (2): the BiLSTMELM-ENSAIT algorithm generally outperformed the Im-BiLSTM model across missing data rates by both RMSE and R^2 values compared to the 'ground truth'.<br><br>At a missing data rate of 50%, the BiLSTMELM-ENSAIT algorithm achieved an RMSE of 0.234 and R^2 of 0.723.<br><br>At a missing data rate of 20%, the BiLSTMELM-ENSAIT algorithm achieved an RMSE of 0.144 and R^2 of 0.794.<br><br>At a missing data rate of 20%, the Im-BiLSTM algorithm achieved an RMSE of 0.209 and R^2 of 0.786.	BiLSTM-ELM has lower RMSE and higher R^2 across multiple datasets and missing rates compare to Im-BiLSTM.	No evaluation of computational cost.<br><br>Small dataset.<br><br>No discussion on average size of artifact (i.e. was it only one point missing or several consecutive points).
Leng et al. [40]	Number and age of participants not reported.<br><br>Eyes open/closed	EEG, sampling rate is not specified (approximated as 127 Hz based on	Missing data is introduced completely at random by randomly removing features from the dataset with rates	14 electrodes using neuroheadset with unspecified location.	The proposed method is SCAPC-GAIN model. The methodology is as follows:<br><br>In the pre-training stage, data with a low missingness rate are imputed first and spectral clustering is used to generate pseudo-labels. Missingness	The method is compared to GAIN [125], PC-GAIN [126], and MA-GAIN [127] models	SCAPC-GAIN had the lowest RMSE across all missing rates, with best performance when parameter β=200. At a missing data rate of	SCAPC-GAIN consistently outperforms existing GAN-based methods in imputing missing EEG	SCAPC-GAIN has a complex structure with many parameters, requiring significant time for

	14,890 timepoints recorded in 117 seconds<br><br>Data available: UCI database.	information provided in article)	from 20% to 80% (step size of 10%).		augmentation is used to ameliorate the model through artificial missingness. These pseudo-labels and the imputed dataset are used to train an auxiliary classifier.<br><br>In the formal training stage, data is used to train both the generator, which models the reconstructed values, and discriminator, which discriminates between true and imputed values, of the model. The pre-trained classifier is integrated into the GAIN framework to guide the generator.<br><br>A 5-fold cross validation is used for evaluation.<br><br>No mention of computer specifications used.		50% the RMSE values for each model were:<br>RMSE (GAIN) = 0.3807<br>RMSE (MA-GAIN) = 0.3979<br>RMSE (PC-GAIN) = 0.3477<br>RMSE (SCAPC-GAIN) = 0.3407<br><br>At a missing data rate of 20% the RMSE values for each model were:<br>RMSE (GAIN) = 0.142<br>RMSE (MA-GAIN) = 0.154<br>RMSE (PC-GAIN) = 0.2107<br>RMSE (SCAPC-GAIN) = 0.124<br><br>However, this difference was not large in magnitude.	data across all missing rates, with the largest improvements observed at extremely high or extremely low missing rates, due to enhanced category-feature learning during pre-training.	parameter tuning during training.<br><br>Subject number, age, sampling rate, and EEG device details are not reported.<br><br>No indication of computational time or amount of data required for functional algorithm.<br><br>No indication of length of missing segments.
Ren and Pan [41]	Two datasets were presented in this article; however, only one was used for missing data interpolation which was:<br><br>Nine subjects<br><br>Performing motor imagery tasks<br><br>No mention of session duration	EEG, sampling rate of 250 Hz, bandpass filtered between 8 and 30 Hz	Missing length in milliseconds tested was 500, 1000 and 2000 .	22-channels placed according to 10-20 system	The proposed model is a TFCMI-CNN-LSTM model for prediction of missing EEG data. The steps of this algorithm are as follows:<br>1. Wavelet transformation is applied on EEG data to obtain time-frequency power information from each channel.<br>2. Time-frequency power information is used to determine linear and non-linear correlations between channels as well as cross mutual information.<br>3. Channels are then organized by similarity using the calculated TFMCI, which is derived using correlation and mutual information.<br>4. Input EEG data and a sequence folding layer serve as the inputs for the	Performance was not compared to any other methodologies	The model performance was evaluated using Spearman Rank Correlation, RMSE, and MAE comparing the true EEG with the interpolated values for channels A01 to A09<br><br>Using no other channels as features, mean values for all channels:<br>RMSE = 6.574<br>MAE = 5.310<br>SR = 0.060	TFCMI-CNN-LSTM model was effectively used to restore abnormal (missing) EEG values.<br><br>Results indicated that using correlation channels as features improved the performance of the model	Fixed values of 500 milliseconds for gaps to interpolate.

	Data available: BCI Competition IV 2a [128]				CNN-LSTM model. The CNN model extracts spatiotemporal features from the data.<br>5. The LSTM model performs time-series prediction, outputting a fully connected layer and a regression layer.<br><br>Did not have any exact train/test split listed.<br><br>No mention of computer specifications used.		Using a random channel as a feature, mean values for all channels:<br>RMSE = 5.847<br>MAE = 4.742<br>SR = 0.305<br><br>Using correlation channels as features, mean values for all channels:<br>RMSE = 3.512<br>MAE = 2.787<br>SR = 0.828	Additional results indicated a slight deterioration in reconstruction accuracy as the missing segment size increase from 500 to 2000 milliseconds.<br><br>The CNN-LSTM model had an average training time of 168.48 seconds where the LSTM model had an average training time of 586.73 seconds.	
Liu et al. (2022) [42]	Three datasets used:<br>(1) 12 participants<br><br>Focusing on stimuli with others present while on stationary bike (either pedalling (1a) or not pedalling (1b)) with stimuli shown at 1 Hz or 2 Hz<br><br>No mention of session duration<br><br>Data available: Bike [49]<br><br>(2) 26 participants	(1) EEG, sampling rate of 512 Hz, bandpass filtered between 1-40 Hz, down sampled from 512 to 128 Hz<br><br>(2) EEG, sampling rate not specified, bandpass filtered between 1-40 Hz, down sampled to128 Hz	Synthesize NaN (missing) values in datasets at rates of 2.5% to 20% (step-size of 2.5%) but results are only displayed for 10%	For (1) and (2): No number or location of electrodes stated.<br><br>For (3): 22 electrodes	This work presents an RNN-based EEG imputation model referred to as SRI-EEG. This model leverages a BiLSTM architecture that uses an input mask to differentiate valid data from artifactual/missing data as well as a temporal decay matrix to incorporate gap duration as a feature to the reconstruction model. This model also incorporates spatial correlations of adjacent EEG channels as well as the state of the trial as additional features.<br><br>Indicated in article that datasets (1) and (2) are split into participant-independent train-test splits of 80% and 20% based on Ding et al. methodology. Also made no specifications regarding how (3) was split.	Performance of the proposed method was compared to: mean value of trial, KNN, SoftImpute which relies on matrix completion via SVD thresholding, ICA (however, it was not clear how ICA was used to fill in gaps in the presence of NaN values), MRNN [129], BRITS [130].	The SRI-EEG outperforms all other algorithms across all datasets by both RMSE and MAE.<br><br>Due to spatial constraints, only results for RMSE (value ± standard deviation) were provided for the top three performing methods for 24 iterations:<br><br>For (1a) at 2Hz:<br>RMSE (SRI-EEG) = 88.192 ± 1.70<br>RMSE (MRNN) = 93.212 ± 1.86	SRI-EEG provides strong performance compared to other methods.<br><br>Task seems to play a role in the accuracy with which the imputation occurs.	Provided vague descriptions of pre-processing methodology followed for the datasets.<br><br>The spatial component relies on the known location and number of electrodes used.<br><br>No mention of computational requirements or latency.<br><br>Reliance on set parameters.

	Performing 'P300 speller task, involving response to flashing letters and numbers<br><br>No mention of session duration<br><br>Data available: Kaggle [50]<br><br>(3) Nine participants<br><br>Motor imagery of hands task<br><br>No mention of session duration<br><br>Data available: BCI Competition IV Dataset 2A [51]	(3) EEG, sampling rate of 250 Hz, bandpass filtered between 0.5 and 100 Hz, down sampled to 128 Hz and bandpass filtered to 1-40 Hz			However, article indicates that 15% of training dataset was used as 'validation'.<br><br>No mention of computer specifications used.		RMSE (BRITS) = 116.477 ± 2.12<br><br>For (1b) at 2Hz:<br>RMSE (SRI-EEG) = 12.142 ± 0.45<br>RMSE (MRNN) = 13.021 ± 0.62<br>RMSE (BRITS) = 13.474 ± 0.51<br><br>For (2):<br>RMSE (SRI-EEG) = 69.985 ± 0.85<br>RMSE (BRITS) = 70.321 ± 1.13<br>RMSE (MRNN) = 72.594 ± 0.72<br><br>For (3):<br>RMSE (SRI-EEG) = 0.889 ± 0.01<br>RMSE (BRITS) = 0.911 ± 0.04<br>RMSE (MRNN) = 0.925 ± 0.03		Unclear training/testing/validation methodology.

AJ-RNN = Adversarial Joint-Learning Recurrent Neural Network, BiLSTM = Bi-directional Long Short-Term Memory Network, BiLSTMELM-ENSAIT = Bi-directional Long Short-Term Memory Network Extreme Learning Machine-Elastic Net self-attention Inception Time Network, BRITS = Bidirectional Recurrent Imputation for Time Series, EEG = Electroencephalogram, GAIN = Generative Adversarial Imputation Networks, ICA = Independent Component Analysis, KNN = k-Nearest Neighbor, MAE = Mean Absolute Error, MA-GAIN = Missingness Augmentation Generative Adversarial Imputation Networks, MRNN = Multi-Directional Recurrent Neural Networks, PC-GAIN = Pseudo-label Conditional Generative Adversarial Imputation Networks, R^2 = Coefficient of Determination, RMSE = Root Mean Square Error, SCAPC-GAIN = Spectral Clustering Augmentation Pseudo-label Conditional Generation Adversarial Imputation Networks, SVD = Singular Value Decomposition

Table S4. Model-Based and Classical Imputation Methods

Reference	Subject information	Signal type	Missing data characteristics	Location and number of sensors	Imputation technique used	Methods compared	Effectiveness	Results of study	Limitations
Ayyoubi et al. [43]	Three patients refractory epilepsy records (33M, 12F, 19F) recorded in the EMU	iEEG signals are recorded using the wireless BIC system (CorTec	Imputation of missing segments of data due to packet loss was a subsection of this article. One of the	Invasive depth electrodes are used in all three participants.	Imputation of missing segments of data due to packet loss was a subsection of this article. The methods that were evaluated included:<br>1. Previous value replacement<br>2. Linear interpolation	Comparison between proposed methods	For patient 1, imputation using PVR elevated the PSD after 100 Hz. The PSD at 500 Hz was -11 dB compared to the raw	Considerable improvement in signal quality can be obtained by employing linear	Reconstruction accuracy is only calculated as PSD between the raw and reconstructed signal. However,

	No mention of task/rest<br><br>Recording durations were 24 hours and 36 minutes for 33M, 23 hours and 37 minutes for 12F, and 24 hours and 17 minutes for 19F<br><br>Data available: N/A	GmbH, Freiburg, Germany) at a sampling rate of 1 kHz as well as clinical devices: Natus Quantum (NQ—Natus Medical Incorporated, Wisconsin, USA) and Nihon Kohden (NK-JE-120-Nihon Kohden Corporation, Tokyo, Japan) at 2 kHz, downsampled to 1 kHz	analysis sections of the paper was investigating how well interpolation methods were able to reconstruct packet loss.<br><br>Random segments were selected from each patient which were measured using the clinical devices and 10% synthetic packet loss.<br><br>Packet loss is described as lost information in wireless transmission of EEG data.	Clinical systems support recording up to 256 channels, but only 32 channels are selected based on the limitations of the BIC system.	3. Spline interpolation<br>These classical interpolation methods are not explained in the article.<br><br>No mention of computer specifications used.		signal which was −33 dB indicating poor imputation and heavy distortion. For patient 2, the PSD at 500 Hz was -23 dB for PVR and -28 dB for raw data. For patient 3, the PSD at 500 Hz was -24 dB compared to -29 dB for raw data. LI and PI showed PSD values close to that of the raw data with an approximate maximum difference of 2 to 3 dB even at 500 Hz.<br><br>For patient 1 using LI and SI, the PSD closely overlaps with the raw signal below 250 Hz. This indicates the accurate recovery of low-frequency content. However, above 250 Hz reconstruction accuracy deteriorates. For patients 2 and 3, these imputation methods had PSD values close to that of the raw data for frequencies below 350 Hz.	imputation in high frequency bands.<br><br>All methods perform well in the low band (<100 Hz).<br><br>Linear imputation and spline imputation perform comparably.	this is an indirect measurement of reconstruction accuracy.<br><br>Real time application and effects on high frequency oscillations are not evaluated.<br><br>Small patient cohort used.
Kanemura et al. [44]	9 healthy participants<br><br>Performing motor imagery tasks.<br><br>288 trials for training and 288 for testing datasets; however, it was unclear as to	EEG sampling rate is 250 Hz, bandpass filtered between 0.5 to 100 Hz with added notch filter at 50 Hz.	Generating missing to stimulate electrode malfunction/movement artifacts.<br><br>Five channels are randomly selected in each trial, and	22 channels are recorded using Ag/AgCl electrodes according to the international 10-20 system.	The proposed method is an MAR-based method for imputation of the missing EEG values. This algorithm considers both previous EEG values as well as concurrently recorded EEG values from other channels to estimate MAR parameters.	The method is compared to NNI and LI methods.	MAR demonstrates the lowest mean of RMSE across the all trial for the nine subjects of the three methods:<br>RMSE (MAR) = 2.143<br>RMSE (NNI) = 3.407<br>RMSE (LI) = 19.333	Iterating the estimation and simulation of the MAR model decreases the imputation errors, and our MAR-based imputation	Impact of imputation on downstream EEG tasks (classification or decoding) is not assessed.<br><br>External validation using a different

	what constituted a "trial"<br><br>Data available: BCI competition IV dataset 2a [128]		data from t=1 to t =1.25 seconds are replaced with NaNs to simulate missing values. 17 of 22 channels were therefore viable at the time of the missing values, enabling the use of both spatial and temporal information for imputation.<br><br>There were 300 sets of EEG data that were corrupted.		The algorithm involves the following: using the testing dataset, nearest neighbor interpolation is initially used to fill in missing values. The MAR model parameters are iteratively determined using the valid signals of the testing dataset as well as the training dataset that is without missing data. A maximum number of 10 iterations were used with 100 MAR parameters being used. A new testing dataset is made using the simulated MAR model. The ability of the model to reconstruct the missing data was evaluated.<br><br>No mention of computer specifications used.		Reconstruction experiments were performed 300 times selecting different missing channels for each patient.	method outperforms the baseline methods.<br><br>RMSE decreases steadily over 10 iterations with no increases, some patients data converged after a few iterations, some took the full 10.	dataset was not provided to evaluate whether this imputation method could function without similar training dataset.
Vafidis et al (2019) [45]	37-year-old female patient with juvenile absence epilepsy<br><br>No mention of task/rest, TMS stimulation occurring<br><br>No mention of session duration<br><br>Data available: N/A	Scalp EEG contaminates by TMS-induced artifacts<br><br>The sampling rate of 1450 Hz and bandpass filtered between 0.1 and 500 Hz.	Induced TMS artifacts were treated as gaps by methods which were included in this review. Original EEG served as "ground truth".<br><br>Artifacts (gaps) were added to (1) resting EEG, (2) at beginning of epileptiform discharge, and (3) during epileptiform discharge.	60 channels in 10-10 montage	Gap filling is a data-driven method used to interpolate missing EEG data based on the assumption that a dynamical system underlies EEG signals and can be extrapolated to reconstruct missing signal. This method uses a local state space model using non-linear time series analysis, searching before and after the gap for segments with similar state-space neighbors to construct a segment with which to reconstruct the missing signal.<br><br>Shape-preserving piecewise cubic spline interpolation used for gap reconstruction, not well explained in the article.<br><br>TMS artifacts were rejected and the gaps that were left by their removal were interpolated.<br><br>FastICA and PCA-based methods were also analyzed in this paper; however, unlike the gap filling and interpolation methods, these methods did not treat	Comparison of four methodologies outlined; however, only gap filling and interpolation methods were considered in this review as the other methods relied on underlying EEG signal to reconstruct.	The ability of these methods to reconstruct missing data was tested both on unfiltered EEG and lowpass filtered at 100 Hz EEG. Unfiltered EEG was the focus for this review, as it better describes the true EEG signal morphology.<br><br>For gap filling method (dataset):<br>RMSE (1) = 13.95<br>RMSE (2) = 20.23<br>RMSE (3) = 44.29<br>NRMSE (1) = 1.353<br>NRMSE (2) = 1.140<br>NRMSE (3) = 2.651<br><br>For shape-preserving piecewise cubic spline interpolation:<br>RMSE (1) = 13.72<br>RMSE (2) = 20.54	Gap filling and shape-preserving piecewise cubic spline interpolation performed similarly in the reconstruction of the EEG signal for (1) and (2).<br><br>Gap filling severely underperforms for (3) likely due to the peaks that occur as a result of seizure.	Gap filling relies upon assumption that state-space properly encapsulates dynamics of EEG signal, shortcomings exist in interpolations during seizures.<br><br>Uses set parameters for gap filling model, not justified why these values were selected.<br><br>Single patient.<br><br>No mention of computational speed or difference between methods.

					the TMS artifacts as gaps and tried to restore the underlying EEG signal.<br><br>No mention of computer specifications used.		RMSE (3) = 18.23<br>NRMSE (1) = 1.333<br>NRMSE (2) = 1.018<br>NRMSE (3) = 1.085		
Xiong et al. (2023) [46]	Three datasets are used:<br>For (1): 100 simulations of TMS-EEG signal<br><br>Number of patients not specified<br><br>No mention of task/rest<br><br>Data sourced from: Alturi et al. [131]<br><br>For (2): 80 “times recording” for each channel<br><br>Data available: Alturi et al. [131]<br><br>For (3): 909.9398 seconds of data<br><br>Data available: figshare public database [132]<br><br>However, datasets 2 and 3 lacked a true ‘ground-truth’, as these signals used resting EEG as the standard for comparison.	For (1) TMS-EEG signals at unspecified sampling rate.<br><br>For (2) TMS-EEG (Neuroscan synnamps2) sampling rate of 20 kHz.<br><br>For (3) TMS-EEG (TMS compatible EEG DC amplifier) at unspecified sampling rate.	Simulated TMS-EEG signals are generated by combining clean EEG and TMS pulse artifact components extracted from public datasets.<br><br>Artifact locations are determined for the proposed algorithm via thresholding for the large amplitude of TMS pulses. The bounds of the artifact window are then determined using derivative thresholding. All those channels affected by the artifact are identified. Baseline correction is performed.	For (1) 64 channels are used at unspecified locations.<br><br>For (2) 3 of 60 channels (1, 37, and 51) are used. Stimulation site is (MNI coordinates: x=-35, y=45, z=38).<br><br>For (3) 64 channels are recorded using 62 c-ring slit electrodes. Stimulation site is (MNI coordinates: x=-20, y=-65, z=65).	This article proposes the 3DARQHI method, which is a signal recombination algorithm based on rational Hermite interpolation.<br><br>The determined artifact locations were removed. The following steps algorithmically reconstructed the signal gap:<br>1. The initial shape parameter is initialized for interpolation using a linear optimization strategy.<br>2. The ARQHI algorithm proposed by Yun et al. [56] is used to interpolate the missing gap.<br>3. The shape parameters are adjusted to an optimal range.<br>4. The output signal is yielded reconstructing the missing gap in all affected channels.<br><br>Computational hardware included: HP Z840 workstation, 16-core Intel Xeon E5-2620 CPU, 32 GB of memory	The method is compared to PCHI [133], PCA [134], FastICA [135], gap filling [45], and SOUND [136] algorithms.<br><br>However, PCA and FastICA did not remove entire signal segments and interpolated, they restored the underlying signal in the presence of the TMS artifacts.<br><br>Testing using: HP Z840 workstation with a 16-core Intel Xeon E5-2620 CPU and 32 G of memory	The effectiveness was measured using SNR, RMSE, and MAE comparing the corrected and raw signals. These metrics were calculated for each method for each dataset.<br><br>The average values for each metric for the original artifact laden signal for dataset 1 were:<br>SNR = -42.20<br>RMSE = 2498.42<br>MAE = 85.35<br><br>The 3DARQHI had the best performance for correction across all metrics compared to the other algorithms, achieving results of:<br>SNR = 30.05<br>RMSE = 0.34<br>MAE = 0.03<br><br>RMSEs for other algorithms include:<br>RMSE(Gap filling) = 19.74<br>RMSE (SOUND) = 4.64<br>PCHI = 1.80<br><br>This algorithm was also the fastest	3DARQHI effectively removes and reconstructs TMS pulse artifacts with better accuracy and speed, outperforming existing methods in both artifact suppression and signal preservation.	3DARQHI requires a standard reference signal (resting EEG) to optimize interpolation parameters.<br><br>Simulated missingness may not represent real world signal loss.<br><br>Method only assesses on TMS pulse artifacts.<br><br>Dataset participant information is not specified.

							processing on a test database constructed of 64 channels of 3-periods (1,200 timepoints) TMS-EEG signals, run 100 times to obtain standard deviation, achieving a mean time of 1.45 seconds, with PCHI at 2.25 seconds, SOUND at 3.54 seconds, and gap filling at 5.86 seconds.		
Kim et al. [47]	Three datasets are used:<br>(1) 3 of 5 patients with spasmodic epilepsy, Subject 1 with eyes open, Subject 3 seizure-free, and Subject 5 with seizure activity<br><br>No mention of task/rest<br><br>23.6 seconds<br><br>Data available: Krug [53]<br><br>(2) Six participants<br><br>Categorization and recognition tasks (100 images)<br><br>1,297,680 timepoints across all trials and participants	For (1) continuous multi-channel EEG (some invasive)<br><br>For (2) EEG.<br><br>For (3) EEG.	Missing data are artificially introduced across all datasets at random rates (5%, 10%, 15% for accuracy analyses) to simulate missingness for testing.	For (1) 100 channels<br><br>For (2) 31 channels are used.<br><br>For (3) 64 channels are used.<br><br>Specific electrode location is not provided for any dataset.	The proposed method is the iEMPCA algorithm. The gaps that have been identified in the EEG data are initially interpolated using the mean value for the previous samples. These estimates are refined using expectation-maximization, which involves updating the PCA basis function and latent components used to estimate the missing values. This involves iteratively updating the number of PCA basis functions used to predict the missing value using energy thresholds with an exponential forgetting factor highlighting more recent EEG trends in the prediction. These eventually converge to a single estimate for the missing data point.<br><br>No mention of computer specifications used.	The method is compared to robust expectation maximization principal component analysis (Robust EMPCA) [57] and missing value singular value decomposition (MSVD) [58].	In dataset (1) iEMPCA has an average RMSE of 0.0344, outperforming Robust EMPCA (0.0431) and MSVD (0.0378).<br><br>In dataset (2) iEMPCA has an RMSE of 0.0163 compared to Robust EMPCA (0.0220) and MSVD (0.0182).<br><br>In dataset (3) iEMPCA underperformed the other methods with an RMSE of 0.0313 compared to MSVD (0.0262) and Robust EMPCA (0.0300).	The method demonstrates faster computation and lower memory use, making it suitable for real time application; however, no data was provided to support this claim, nor information regarding the CPU used for the test.<br><br>Indicated that multivariate EEG could be described using only a few PCA basis functions, reducing memory complexity.	No discussion as to why iEMPCA outperformed other methods in two datasets but not the third.

	Data available: Event related potential dataset [54]<br><br>(3) Three participants<br><br>Performing task needing to target computer cursor<br><br>1-6 sessions each participant, totalling 520.704 timepoints<br><br>Data available: Self-regulation EEG data by Klaus [55]								

3DARQHI = 3D adaptive rational quadratic Hermite interpolation, ADMM = Alternating Direction Method of Multipliers, EM = Expectation Maximization, EMPCA = Expectation Maximization Principal Component Analysis, iEMPCA = Incremental Expectation Maximization Principal Component Analysis, ICA = Independent Component Analysis, LGS = Local Graph Signal Smoothness, LI = Linear Interpolation, MAR = Multivariate Autoregressive, MNI = Montreal Neurological Institute coordinate system, MSVD = Missing Value Singular Value Decomposition, NMSE = Normalized Mean Squared Error, NNI = Nearest-Neighbor Interpolation, NRMSE = Normalized Root Mean Square Error, PCA = Principle Component Analysis, PCA = Principal Component Analysis, PCHI = Piecewise Cubic Hermite Interpolation, PSD = Power Spectral Density, PVR = Previous Value Replacement, RCLR = Robust Cauchy based Low-Rank model, RMSE = Root Mean Square Error, RMSE = Root Mean Squared Error, SI = Spline Interpolation, SNR = Signal to Noise Ratio, SOUND = Structured Output Unsupervised Denoising, SSI = Spherical Spline Interpolation, SVD = Singular Value Decomposition, TMS = Transcranial Magnetic Stimulation

110. BSIRIKEN, Japan; Shanghai Jiao Tong University, China Advanced Brain Signal Processing
111. Cichocki, A.; Mandic, D.; Phan, A.-H.; Caiafa, C.; Zhou, G.; Zhao, Q.; De Lathauwer, L. Tensor Decompositions for Signal Processing Applications From Two-Way to Multiway Component Analysis. **2014**, doi:10.48550/ARXIV.1403.4462.
112. Acar, E.; Dunlavy, D.M.; Kolda, T.G.; Mørup, M. Scalable Tensor Factorizations for Incomplete Data. *Chemometrics and Intelligent Laboratory Systems* **2011**, *106*, 41–56, doi:10.1016/j.chemolab.2010.08.004.
113. Caiafa, C.F.; Cichocki, A. Multidimensional Compressed Sensing and Their Applications. *WIREs Data Min & Knowl* **2013**, *3*, 355–380, doi:10.1002/widm.1108.
114. Zhao, Q.; Zhang, L.; Cichocki, A. Bayesian CP Factorization of Incomplete Tensors with Automatic Rank Determination. **2014**, doi:10.48550/ARXIV.1401.6497.
115. Boyd, S. Distributed Optimization and Statistical Learning via the Alternating Direction Method of Multipliers. *FNT in Machine Learning* **2010**, *3*, 1–122, doi:10.1561/2200000016.
116. Liu, J.; Musialski, P.; Wonka, P.; Ye, J. Tensor Completion for Estimating Missing Values in Visual Data. *IEEE Trans. Pattern Anal. Mach. Intell.* **2013**, *35*, 208–220, doi:10.1109/TPAMI.2012.39.
117. Leeb, R.; Lee, F.; Keinrath, C.; Scherer, R.; Bischof, H.; Pfurtscheller, G. Brain-Computer Communication: Motivation, Aim, and Impact of Exploring a Virtual Apartment. *IEEE transactions on neural systems and rehabilitation engineering* **2007**, *15*, 473–482, doi:10.1109/TNSRE.2007.906956.
118. Schlögl, A. Outcome of the BCI-Competition 2003 on the Graz Data Set.
119. Zhang, Y.; Zhou, G.; Zhao, Q.; Cichocki, A.; Wang, X. Fast Nonnegative Tensor Factorization Based on Accelerated Proximal Gradient and Low-Rank Approximation. *Neurocomputing* **2016**, *198*, 148–154, doi:10.1016/j.neucom.2015.08.122.
120. Ofner, P.; Schwarz, A.; Pereira, J.; M&uuml, G.R.; ller-Putz Upper Limb Movements Can Be Decoded from the Time-Domain of Low-Frequency EEG. *PLoS ONE* **2017**, *12*, e0182578–e0182578, doi:10.1371/journal.pone.0182578.
121. Yi-Lei Chen; Chiou-Ting Hsu; Liao, H.-Y.M. Simultaneous Tensor Decomposition and Completion Using Factor Priors. *IEEE Trans. Pattern Anal. Mach. Intell.* **2014**, *36*, 577–591, doi:10.1109/TPAMI.2013.164.
122. EEGEpilepsyDatasets.

123. Chen, B.; Zheng, H.; Wang, L.; Hellwich, O.; Chen, C.; Yang, L.; Liu, T.; Luo, G.; Bao, A.; Chen, X. A Joint Learning Im-BiLSTM Model for Incomplete Time-Series Sentinel-2A Data Imputation and Crop Classification. *International Journal of Applied Earth Observation and Geoinformation* **2022**, *108*, 102762, doi:10.1016/j.jag.2022.102762.
124. Ma, Q.; Li, S.; Cottrell, G.W. Adversarial Joint-Learning Recurrent Neural Network for Incomplete Time Series Classification. *IEEE Transactions on Pattern Analysis and Machine Intelligence* **2022**, *44*, 1765–1776, doi:10.1109/TPAMI.2020.3027975.
125. Yoon, J.; Jordon, J.; van der Schaar, M. GAIN: Missing Data Imputation Using Generative Adversarial Nets 2018.
126. Wang, Y.; Li, D.; Li, X.; Yang, M. PC-GAIN: Pseudo-Label Conditional Generative Adversarial Imputation Networks for Incomplete Data 2020.
127. Wang, Y.; Li, D.; Xu, C.; Yang, M. Missingness Augmentation: A General Approach for Improving Generative Imputation Models 2021.
128. BCICIV_2a_gdf.
129. Yoon, J.; Zame, W.R.; van der Schaar, M. Multi-Directional Recurrent Neural Networks: A Novel Method for Estimating Missing Data. In Proceedings of the Time Series Workshop; Sydney; pp. 1–5.
130. Cao, W.; Wang, D.; Li, J.; Zhou, H.; Li, L.; Li, Y. BRITS: Bidirectional Recurrent Imputation for Time Series 2018.
131. Atluri, S.; Frehlich, M.; Mei, Y.; Garcia Dominguez, L.; Rogasch, N.C.; Wong, W.; Daskalakis, Z.J.; Farzan, F. TMSEEG: A MATLAB-Based Graphical User Interface for Processing Electrophysiological Signals during Transcranial Magnetic Stimulation. *Front. Neural Circuits* **2016**, *10*, doi:10.3389/fncir.2016.00078.
132. Rogasch, N.C.; Sullivan, C.; Thomson, R.H.; Rose, N.S.; Bailey, N.W.; Fitzgerald, P.B.; Farzan, F.; Hernandez-Pavon, J.C. Analysing Concurrent Transcranial Magnetic Stimulation and Electroencephalographic Data: A Review and Introduction to the Open-Source TESA Software. *NeuroImage* **2017**, *147*, 934–951, doi:10.1016/j.neuroimage.2016.10.031.
133. Gabrielides, N.C.; Sapidis, N.S. Cubic Polynomial and Cubic Rational C 1 Sign, Monotonicity and Convexity Preserving Hermite Interpolation. *Journal of Computational and Applied Mathematics* **2019**, *357*, 184–203, doi:10.1016/j.cam.2019.02.024.
134. ter Braack, E.M.; de Jonge, B.; van Putten, M.J.A.M. Reduction of TMS Induced Artifacts in EEG Using Principal Component Analysis. *IEEE Trans Neural Syst Rehabil Eng* **2013**, *21*, 376–382, doi:10.1109/TNSRE.2012.2228674.
135. Rogasch, N.C.; Thomson, R.H.; Farzan, F.; Fitzgibbon, B.M.; Bailey, N.W.; Hernandez-Pavon, J.C.; Daskalakis, Z.J.; Fitzgerald, P.B. Removing Artefacts from TMS-EEG Recordings Using Independent Component Analysis: Importance for Assessing Prefrontal and Motor Cortex Network Properties. *NeuroImage* **2014**, *101*, 425–439, doi:10.1016/j.neuroimage.2014.07.037.
136. Mutanen, T.P.; Metsomaa, J.; Makkonen, M.; Varone, G.; Marzetti, L.; Ilmoniemi, R.J. Source-Based Artifact-Rejection Techniques for TMS–EEG. *Journal of Neuroscience Methods* **2022**, *382*, 109693, doi:10.1016/j.jneumeth.2022.109693.
